# AP2XII-1 and AP2XI-2 Suppress Schizogony Gene Expression in *Toxoplasma gondii*

**DOI:** 10.3390/ijms25105527

**Published:** 2024-05-18

**Authors:** Yucong Jiang, Yuehong Shi, Yingying Xue, Dandan Hu, Xingju Song

**Affiliations:** 1 College of Animal Science and Technology, Guangxi University, Nanning 530004, China; jiangyucong0401@163.com (Y.J.); xueyingying1204@163.com (Y.X.); hudandan@gxu.edu.cn (D.H.); 2 Guangxi Zhuang Autonomous Region Engineering Research Center of Veterinary Biologics, Nanning 530004, China; shiyuehong1223@163.com; 3 Guangxi Key Laboratory of Animal Breeding, Disease Control and Prevention, Nanning 530004, China

**Keywords:** *Toxoplasma gondii*, ApiAP2, conditional knockdown, merozoite, MORC

## Abstract

*Toxoplasma gondii* is an intracellular parasite that is important in medicine and veterinary science and undergoes distinct developmental transitions in its intermediate and definitive hosts. The switch between stages of *T. gondii* is meticulously regulated by a variety of factors. Previous studies have explored the role of the microrchidia (MORC) protein complex as a transcriptional suppressor of sexual commitment. By utilizing immunoprecipitation and mass spectrometry, constituents of this protein complex have been identified, including MORC, Histone Deacetylase 3 (HDAC3), and several ApiAP2 transcription factors. Conditional knockout of MORC or inhibition of HDAC3 results in upregulation of a set of genes associated with schizogony and sexual stages in *T. gondii* tachyzoites. Here, our focus extends to two primary ApiAP2s (AP2XII-1 and AP2XI-2), demonstrating their significant impact on the fitness of asexual tachyzoites and their target genes. Notably, the targeted disruption of AP2XII-1 and AP2XI-2 resulted in a profound alteration in merozoite-specific genes targeted by the MORC–HDAC3 complex. Additionally, considerable overlap was observed in downstream gene profiles between AP2XII-1 and AP2XI-2, with AP2XII-1 specifically binding to a subset of ApiAP2 transcription factors, including AP2XI-2. These findings reveal an intricate cascade of ApiAP2 regulatory networks involved in *T. gondii* schizogony development, orchestrated by AP2XII-1 and AP2XI-2. This study provides valuable insights into the transcriptional regulation of *T. gondii* growth and development, shedding light on the intricate life cycle of this parasitic pathogen.

## 1. Introduction

*Toxoplasma gondii* is an intracellular protozoan parasite belonging to the phylum Apicomplexa. It poses a significant risk to pregnant women and immunocompromised individuals, causing severe complications and constituting a public health concern worldwide [1]. The eukaryotic parasite *T. gondii* undergoes very different developmental patterns in its intermediate and definitive hosts. In intermediate hosts such as humans and other warm-blooded animals, *T. gondii* undergoes a process known as asexual endodyogeny. During acute infections, rapidly dividing tachyzoites are responsible for the active replication and dissemination of the parasite. In chronic infections, bradyzoites form tissue cysts that can persist within the host for long periods of time, contributing to the establishment of chronic infections [2]. However, when *T. gondii* infects the intestines of definitive-host felines, it undergoes a distinct coccidian-like life cycle. This life cycle involves both asexual and sexual development, leading to the production of specialized stages called oocysts. These oocysts, which are shed into the feces of infected felines, represent a significant source of infection in other animals and play a key role in the transmission of *T. gondii*. Understanding the complex life cycle and developmental processes of *T. gondii* is crucial for elucidating the mechanisms of infection and transmission as well as for developing effective strategies to control this disease. Previous experiments have provided evidence that environmental changes can induce stage shifts in *T. gondii*. For instance, exposure to an alkaline medium has been shown to trigger the transition from tachyzoites to bradyzoites, i.e., the dormant stage of the parasite [3]. Furthermore, the addition of linoleic acid has been found to promote the commitment of cell-cultured parasites to sexual development, a process typically restricted to the intestinal environment of the definitive host [4,5]. These findings highlight the sensitivity of *T. gondii* to its environment and its ability to adapt its life cycle accordingly. Moreover, extensive studies have revealed distinct gene expression patterns and chromatin remodeling events that occur at each stage of the parasitic life cycle [6,7]. These observations suggest the involvement of intricate epigenetic regulatory mechanisms in orchestrating stage transitions in *T. gondii*.

Epigenetic modifications, such as DNA methylation and histone modifications, play pivotal roles in regulating gene expression and contributing to the phenotypic plasticity of the parasite [8]. Recently, Farhat et al. [9] identified a protein complex that acts as a transcriptional repressor of sexual commitment [9]. This protein complex consists of a microrchidia (MORC), histone deacetylase 3 (HDAC3), and several Apicomplexan AP2 (ApiAP2) transcription factors that can be identified by immunoprecipitation (IP) and mass spectrometry [9]. The ApiAP2 family proteins encompass at least one AP2 domain, can bind specifically to DNA, and can regulate the related biological process. Conditional knockdown of MORC or inhibition of HDAC3 results in a large number of sexual-stage-specific genes expressed in tachyzoites. A MORC/HDAC3-associated cascading ApiAP2 transcriptional regulation network was then presented in the transition of developmental trajectories [9]. These findings provide compelling evidence for the involvement of MORC/HDAC3/ApiAP2s in regulating the transition of developmental trajectories in *T. gondii*.

As a homolog of the plant Apetela2/ethylene response factor (AP2/ERF), ApiAP2 contains a plant-like DNA-binding domain that can act as either an activator or a suppressor of gene transcription [10,11,12,13,14]. Despite the unveiling of the gene silencing role of the MORC–HDAC3–ApiAP2 complex, the specific cluster of genes regulated by the binding of specific ApiAP2s in its promoter remains largely unknown. Current studies have shown that AP2XII-1 and AP2XI-2 bind to each other together with MORC and HDAC3, and the depletion of these two transcription factors results in presexual gene expression and merogony development [15,16,17]. As a concurrent study, we characterized the two primary ApiAP2s from the MORC–HDAC3–ApiAP2 complex based on its phenotype score [18], and found that they are all important for tachyzoite growth and development. Conditional knockdown of AP2XII-1 or AP2XI-2 resulted in MORC/HDAC3-targeted merozoite-specific gene alterations. APXII-1 and AP2XI-2 showed highly overlapping downstream gene repertoires, and AP2XII-1 could specifically bind to the promoters of a set of ApiAP2s, including AP2XI-2. Our results suggest that there is a sophisticated cascading ApiAP2 regulation network in *T. gondii* sexual development, coordinately derived from AP2XII-1 and AP2XI-2.

## 2. Results

### 2.1. A Strong Correlation between the Downstream Promoters Bound by AP2XII-1 and AP2XI-2 and MORC

**Figure 1:**
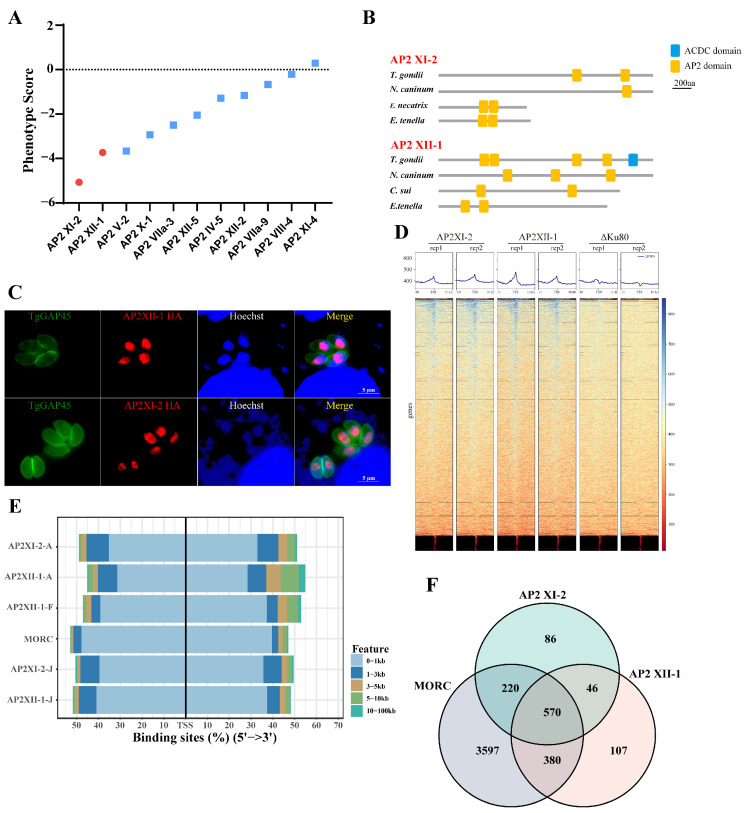
Target genes of AP2XII-1 and AP2XI-2 showing a high degree of overlap with MORC. (**A**) Phenotype scores [18] of ApiAP2 transcription factors in MORC immunoprecipitation products. Red circles represent the transcription factors investigated in this study. (**B**) Schematic representation of conserved AP2-domain-containing proteins from *T. gondii* (TGGT1_218960, TGGT1_310900), *Neospora caninum* (NCLIV_061420, NCLIV_054920), *Cystoisospora suis* (CSUI_000869), *Eimeria tenella* (ETH2_0940300, ETH2_0734800), and *Eimeria necatrix* (ENH_00027630). Domains were predicted by SMART (http://smart.embl-heidelberg.de/, accessed on 18 March 2022). The yellow square represents the AP2 domain and the blue square represents the ACDC domain. (**C**) Subcellular localization of AP2XII-1 and AP2XI-2 proteins in the parasites. Endogenously tagged parasites were co-stained with mouse anti-HA (red) and rabbit anti-GAP45 (green), and nuclei were stained with Hoechst (blue). Scale bars = 5 μm. (**D**) Profile and heatmaps of the averaged sum showing the Cut-Tag called the peaks of AP2XII-1 (HA) and AP2XI-2 (HA) around the TSS of the parasite genes. The top panels show the average signal profile of the genomic loci centered on the TSS (±10 kb). The lower panels show heatmaps of the peak density around the same genomic loci. The color scale used to interpret signal intensity is located on the right side of each graph. (**E**) Genome-wide peak occupancy profiles for MORC, AP2XII-1, and AP2XI-2 proteins. The average signal profiles of each protein are plotted across the 0 kb to 100 kb region relative to the transcription initiation site of the nearest *T. gondii* gene. AP2XII-1-F represents the data from Fan et al. [16] and AP2XII-1-A and AP2XI-2-A are data from Antunes et al. [15]. (**F**) Venn diagram analysis of peaks for MORC (n = 4767), AP2XII-1 (n = 1103), and AP2XI-2 (n = 922).

MORC is thought to control the development of *T. gondii* by driving transcriptional switches [9]. In this study, we focused on two ApiAP2 transcription factors: AP2XII-1 (TGGT1_218960) and AP2XI-2 (TGGT1_310900), which had the lowest phenotypic scores among the ApiAP2 factors immunoprecipitated with MORC in previous studies [9] (Figure 1A). Comparative analysis of the sequence architecture and homologs of these two AP2 factors revealed that AP2XII-1 and AP2XI-2 have a unique architecture and showed less similarity to other organisms (Figure 1B and Figure S1A,B). To examine specific gene clusters regulated downstream by AP2XII-1 and AP2XI-2, a Cut-Tag technique using epitope-tagged parasites was employed. We strategically fused the mAID-3HA tag to the C-terminus of the respective endogenous genes to enable tagging under their native promoters (Figure S2A–C). Immunofluorescence assays revealed the nuclear localization of AP2XII-1 and AP2XI-2, with stable expression (except for AP2XII-1, which had higher expression in the S/M phase) observed at all phases of the tachyzoite cell cycle (Figure 1C and Figure S3A–D). Cut-Tag results demonstrate that both AP2XII-1 and AP2XI-2 exhibited a high degree of enrichment at the transcription start site (TSS) (Figure 1D,E, Tables S1 and S2), which is consistent with the results of Fan et al. [16] and Antunes et al. [15]. Interestingly, despite these distinctions, the downstream genes regulated by AP2XII-1 and AP2XI-2 significantly overlapped with those regulated by MORC (Figure 1F), which is also highly consistent with the targets of AP2XII-1, AP2XI-2, and AP2XII-2 in recent studies (Figure S4A–C) [15,16]. Hence, we speculate that the downstream genes regulated by AP2XII-1 and AP2XI-2 are highly correlated with MORC.

### 2.2. AP2XII-1 and AP2XI-2 Are Critical for Tachyzoite Replication In Vitro

**Figure 2:**
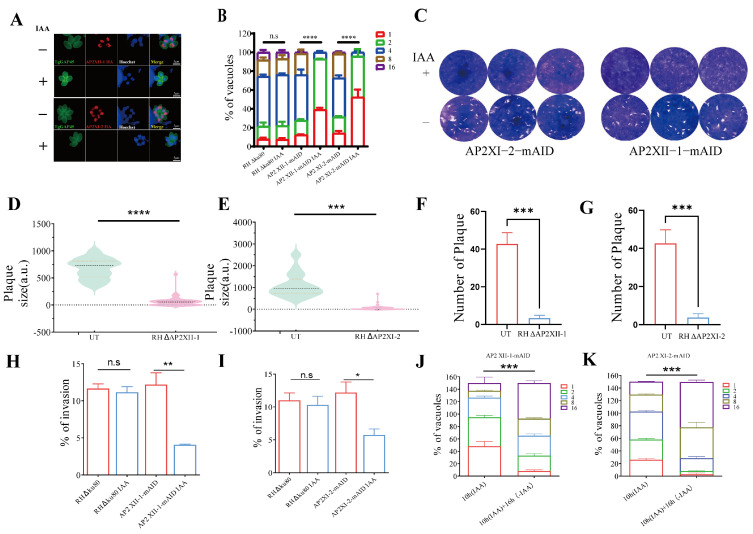
AP2XII-1 and AP2XI-2 are essential for the replication of *T. gondii* tachyzoites. (**A**) Fluorescence microscopy of intracellular AP2XII-1-mAID-HA and AP2XI-2-mAID-HA parasites after treatment with 500 μM IAA or a vehicle (right panel). Tachyzoites were co-stained with mouse anti-HA (red) and rabbit anti-TgGAP45 (green) antibodies. Nuclei were stained with Hoechst (blue). Scale bars = 5 μm. ZEN 3.0 software was used to calculate the mean fluorescence intensity (right panel) of these proteins. ****, *p* < 0.0001. (**B**) Intracellular parasite replication after 24 h of post-infection incubation with IAA or a vehicle. Data are expressed as the mean ± SEM of three independent assays, with 100 total PVs of each strain counted for each assay. (**C**) Plaque assay of AP2XII-1-mAID-3HA and AP2XI-2-mAID-3HA parasites grown for 7 days on IAA- or vehicle-treated HFF monolayers. The plaque areas (**D**,**E**) and plaque numbers (**F**,**G**) were measured and counted by Photoshop CC 2019 software: 20.0.4 20190227.r.76 2019/02/27: 1205725 x64 (Adobe, San Francisco, CA, USA). (**H**,**I**) The invading efficiency of AP2XII-1 and AP2XI-2 knockdown parasites. HFFs were inoculated with 1 × 10^5^ parasites and continuously cultured for 24 h. The invasion ratio is expressed as the number of vacuoles per host cell. (**J**,**K**) The recovered replication ability of AP2XII-1 and AP2XI-2 knockdown parasites. Parasites were treated with IAA for 10 h, then the IAA was removed and the parasites were cultured for an additional 16 h. The number of tachyzoites in the PV was counted by three independent assays of 100 PV each. *, *p* < 0.05; **, *p* < 0.01; ***, *p* < 0.001; ****, *p* < 0.0001; n.s, no significant difference.

The phenotype scores [18] of AP2XII-1 and AP2XI-2 were −3.73 and −5.07, respectively. This strongly suggests that they may be genes essential for parasite growth. Consequently, an indole-3-acetic acid (IAA)-induced degradation (mAID) strategy [19] was performed to conditionally knockdown AP2XII-1 and AP2XI-2 proteins by the addition of IAA (Figure S2A). Immunofluorescence assays (IFAs) and Western blots revealed that the addition of IAA for 20 h led to the degradation of AP2XII-1 and AP2XI-2 proteins in the parasite (Figure 2A and Figure S5A,B). To investigate the role of AP2XII-1 and AP2XI-2 during the parasite’s lytic cycle, a growth phenotype analysis was conducted. Upon IAA treatment and subsequent knockdown of AP2XII-1 and AP2XI-2, the parasites were unable to form plaques in host cells (Figure 2C). In comparison with the gene knockdown strains, the parental strain exhibited a significantly greater plaque area (Figure 2D,E) and a greater number of plaques formed within the cells (Figure 2F,G). Further analysis demonstrated that degradation of AP2XII-1 and AP2XI-2 resulted in significant defects in parasite replication and invasion. Their replication ability was significantly reduced, with mostly one or two tachyzoites per vacuole and rarely more than four tachyzoites (Figure 2B). Moreover, their invasive capacity was diminished (Figure 2H,I). However, parasite replication defects resulting from the degradation of AP2XII-1 and AP2XI-2 proteins were not irreversible. After treatment with IAA for 10 h to induce the degradation of AP2XII-1 and AP2XI-2 proteins, parasites continued to be cultured for an additional 16 h in the absence of IAA [20]. It was observed that the proliferation ability of these two genetically edited parasites had significantly recovered during this period (Figure 2J,K).

### 2.3. The Transcriptional Levels of Downstream Genes Regulated by AP2XII-1 Were Correlated with MORC

**Figure 3:**
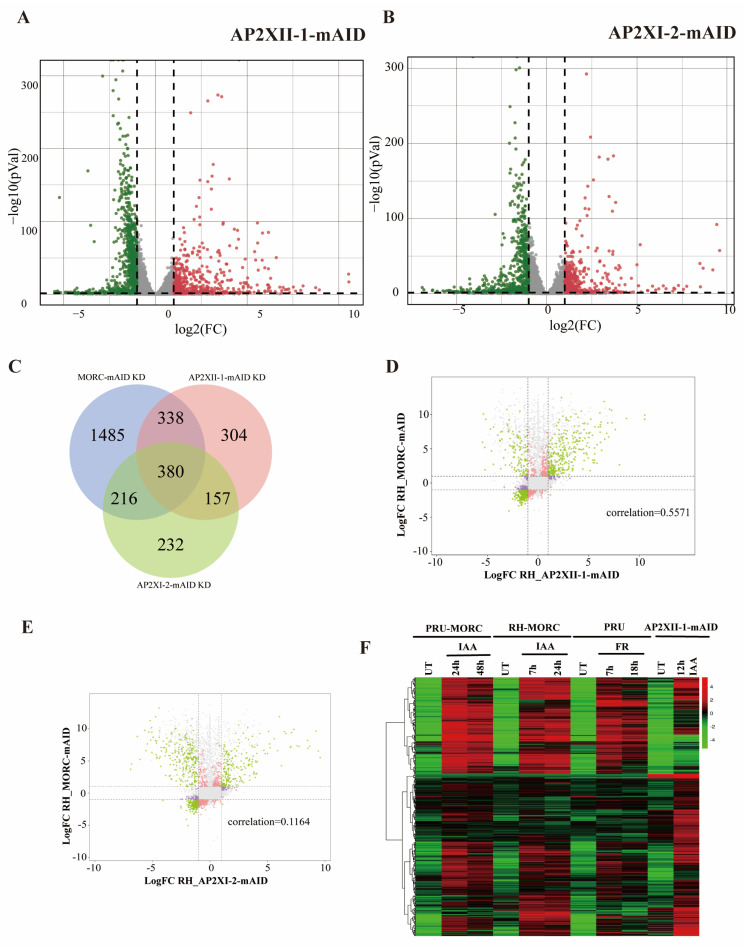
Genes regulated by AP2XII-1, AP2XI-2, and MORC are highly overlapping. (**A**,**B**) Volcano plots showing differentially expressed genes after gene knockdown by IAA treatment for 12 h (n = 8920). Green and red dots indicate significantly downregulated and upregulated genes, respectively, and gray dots indicate genes that are not significantly different. (**C**) Venn diagram analysis of differentially expressed genes in parasites after knockdown of AP2XII-1 (n = 1179), AP2XI-2 (n = 985), and MORC (n = 2419). (**D**,**E**) Pearson correlation between gene alterations after the knockdown of MORC and AP2s. Pearson correlation coefficients (r) are given. Green dots represent positive correlated DEGs, while the rest dots shows no correlation. (**F**) Heatmap showing genes upregulated after AP2XII-1 or MORC [9] knockdown. The color scale indicates (Log2 + 1)-transformed fold changes.

To identify genes that may be regulated by AP2XII-1 and AP2XI-2, RNA-seq analysis was conducted. AP2XII-1-deficient parasites displayed 1179 differentially expressed genes, of which 459 were upregulated genes and 720 were downregulated genes (Figure 3A, Table S3). Similarly, AP2XI-2-deficient parasites exhibited 985 differentially expressed genes, including 387 upregulated and 598 downregulated genes (Figure 3B, Table S4).

Considering that AP2XII-1 and AP2XI-2 were detected in MORC immunoprecipitation products [9], we investigated whether there was any overlap in the downstream genes regulated by them. Interestingly, Venn diagram analysis revealed that 60.9% (718/1179) and 60.5% (596/985) of the differentially expressed genes in AP2XII-1 and AP2XI-2 knockdown parasites overlapped with those in MORC knockdown parasites, respectively (Figure 3C). Correlation analysis was performed to explore the relationship between the two ApiAP2s and MORC. AP2XII-1-regulated genes showed a moderate degree of correlation (Pearson’s correlation coefficient = 0.5571) with MORC-regulated genes (Figure 3D), while AP2XI-2 showed a lower correlation with MORC (a Pearson’s correlation coefficient of 0.1164) (Figure 3E). Heatmap analysis revealed that the regulatory trends of downstream genes by AP2XII-1 and AP2XI-2 were significantly similar to those of MORC, and, in particular, the upregulated genes in AP2XII-1-deficient strains showed a highly consistent trend with that of MORC (Figure 3F and Figure S6A–C).

### 2.4. AP2XII-1 and AP2XI-2 Deficiency Induces the Expression of EES-Restricted Transcripts and Parasite Endopolygeny

**Figure 4:**
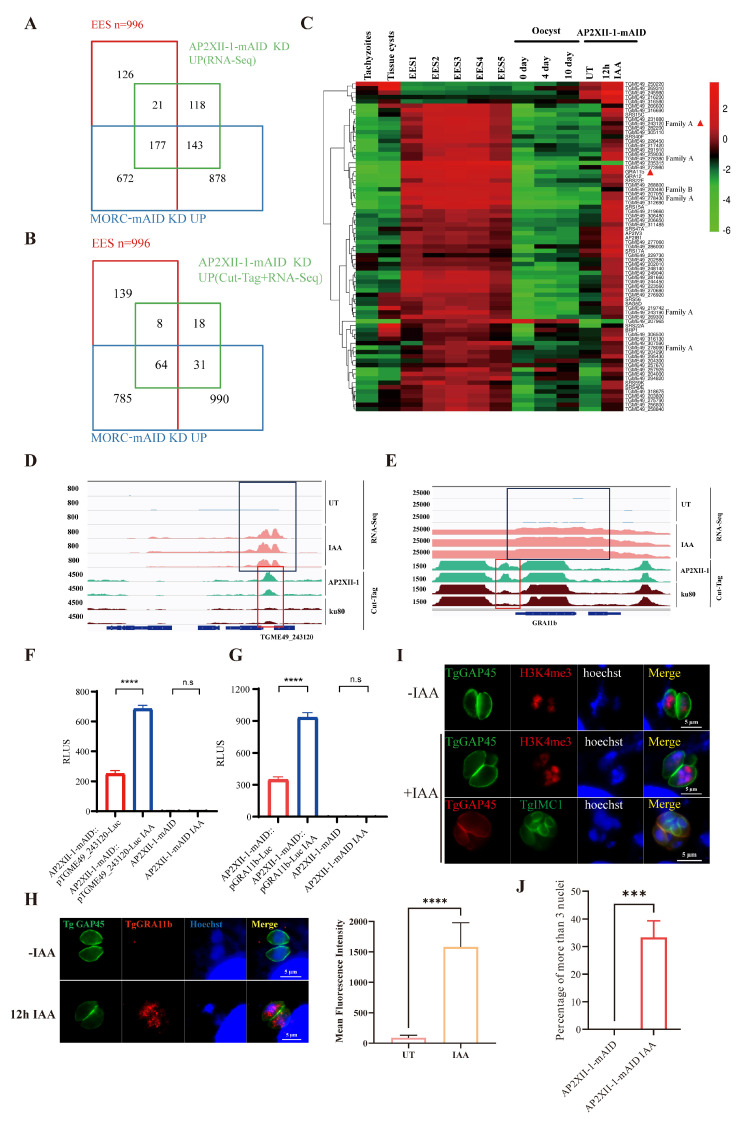
Depletion of AP2XII-1 induces the expression of enteroepithelial-stage-restricted genes. Venn diagram of genes upregulated after AP2XII-1 knockdown. (**A**) (n = 459 genes) and (**B**) (n = 121 genes) show Cut-Tag peaks of AP2XII-1 against the EES-specific genes [22] and the upregulated genes after MORC knockdown [9] (n = 1870 genes). (**C**) Hierarchical clustered transcriptional heatmap showing highly upregulated genes (Log2FC ≥ 2, RNA-seq) after AP2XII-1 depletion and AP2XII-1-bounded targets (Cut-Tag data). The transcript abundance at various developmental stages, namely the tachyzoite, bradyzoite/cyst, EES, and oocyst stages, is displayed. Triangles represent genes of interest. The color scale indicates (Log2 + 1)-transformed fold changes. The red triangle represents the genes we’re interested in. (**D**,**E**) IGV screenshots of two genomic regions with representative merozoite genes. Cut-Tag profiles were obtained using antibodies directed against HA (AP2XII-1-tagged) in chromatin sampled from the AP2XII-1-mAID-HA strain and the Δku80 strain. RNA-seq data from AP2XII-1-mAID-HA parasites after 12 h of treatment or no treatment with IAA are shown in red and blue peaks. The normalized RPKMs for Cut-Tag and RNA-seq reads are shown on the *y*-axis. The red box indicates the AP2XII-1-enriched DNA peaks and the black box indicates the mRNA levels of the target genes (GRA11b and TGME49_243120). Independent repeats are shown. (**F**,**G**) Relative RLUs of AP2XII-1-mAID-HA parasites expressing luciferase under the control of GRA11b (**F**) or TGME49_243120 (**G**) promoter. pGRA11b-Luc::AP2XII-1-mAID, pTGME49_243120-Luc::AP2XII-1-mAID, and AP2XII-1-mAID were treated with IAA or a vehicle for 12 h and then detected for luciferase expression. (**H**) Immunofluorescence analysis of GRA11b expression in AP2XII-1 knockdown parasites. Intracellular AP2XII-1-mAID-HA parasites were treated with 500 μM IAA or a vehicle for 12 h, then the tachyzoites were co-stained with mouse anti-GRA11b (red) and rabbit anti-TgGAP45 (green) antibodies. Nuclei were stained with Hoechst (blue). The scale bar is 5 μm. ****, *p* < 0.0001; n.s, no significant difference. ZEN 3.0 software was used to calculate the mean fluorescence intensity (right panel) of the GRA11b protein expressed after AP2XII-1 knockdown. ****, *p* < 0.0001. (**I**) IFA showing the endopolygeny division of the AP2XII-1 knockdown strain. Note that many IAA-treated parasites contain three or more daughter cells budding and the image of a merozoite delineated by TgGAP45 (green) showing polyploidy (n = 4). The nuclear structure was co-stained with Hoechst DNA-specific dye and H3K4me3. (**J**) Statistics of multi-nucleus parasites (n ≥ 3) formed after AP2XII-1 knockdown. Parasites were treated with IAA for 12 h. The number of nuclei was determined by three independent assays of 100 parasites each. ***, *p* < 0.001.

Previous studies have shown that the MORC–HDAC3–ApiAP2 protein complex acts as a master regulator of gene transcription switches and developmental transitions in *T. gondii* [9]. Considering the correlation between AP2XII-1 and MORC (Figure 3E), transcriptomic data from the bradyzoites [21], merozoites [22], enteroepithelial stage (EES) [23], and oocysts [24] were used to analyze the developmental stages regulated by AP2XII-1. The EES is the coccidian-like developmental stage of *T. gondii* in the cat’s intestinal epicellular cells, including asexual schizogony, sexual commitment/gametocytogenesis, and the development of zygotes and unsporulated oocysts [23,25,26]. Our results reveal that AP2XII-1 upregulated the expression of 198 EES genes (including merozoite and sexual-stage-specific genes), accounting for 30% (198/459) of the upregulated genes caused by AP2XII-1 knockdown (Figure 4A, Table S5). These results are consistent with previous studies by Fan et al. [16] and Antunes et al. [15] (Figure S7A,B). There were few genes specific to the sporozoite (15/295) and bradyzoite (42/331) stages [24,27] (Figure S8A,B). Combined with previous Cut-Tag data for the AP2XII-1 protein, we found that 121 of the 459 upregulated genes had promoters capable of being bound and regulated by the AP2XII-1 protein (Figure 4B, Table S6). Most of these genes were specific to the EES, including merozoite markers such as GRA11b [28] and a number of *T. gondii* family A proteins, which are predicted to be merozoite-specific secretions and/or membrane-associated proteins [22] (Figure 4C). AP2XII-1 was also observed to bind the promoter regions of these EES-specific genes, including TGME49_237800 and GRA11b (Figure 4D,E and Figure S9A–E). Additionally, numerous merozoite-restricted surface proteins (SRSs) were found to be upregulated after AP2XII-1 KD (Figure S10A, Table S7), which was also observed in MORC knockdown *T. gondii* strains [9] (Figure S10B).

To further validate the expression of EES-specific genes in cell-cultured tachyzoites, we constructed a luciferase reporter system to characterize the regulatory role of AP2XII-1 in the promoter of its target genes in AP2XII-1-mAID-HA parasites. A firefly luciferase gene was inserted into the TgUPRT locus of the AP2XII-1-mAID parasite strain by CRISPR/Cas9 technology (Figure S11A,B). The promoter of the firefly luciferase gene was replaced with the promoter sequence of GRA11b or TGME49_243120 (*T. gondii* family A protein), thus bringing the expression of the luciferase gene under the regulatory control of the GRA11b or TGME49_243120 promoters. After 12 h of IAA treatment, luciferase expression was significantly elevated in AP2XII-1-mAID::pGRA11b-Luc and AP2XII-1-mAID::p TGME49_243120-Luc parasites compared with untreated parasites (Figure 4F,G). Meanwhile, the accumulation of merozoite-specific GRA11b proteins was confirmed in AP2XII-1-mAID parasites by immunofluorescence assay using mouse anti-GRA11b monoclonal antibodies after 12 h of IAA treatment (Figure 4H).

Disruption of MORC or HDAC3 resulted in the transition of parasite division from endodyogeny to endopolygeny. Here, we wanted to know if AP2XII-1 deficiency could also result in endopolygeny. Immunofluorescence staining with nuclei (Hoechst dye and H3K4me3), inner membrane complex (TgIMC1), and outer membrane (TgGAP45) markers was performed to distinguish endodyogeny and endopolygeny. Our results show that, in AP2XII-1-mAID-HA parasites, typical endodyogeny division with a mother cell contains a maximum of two nuclei or daughter cells without IAA treatment (Figure 4I), while about 30% of the parasites showed characteristics of endopolygeny with multiple (more than two) nuclei and daughter cells within an individual mother cell after 12 h of IAA treatment (Figure 4I,J). These results suggest that AP2XII-1 in the MORC–HDAC3–ApiAP2 protein complex is crucial for the parasite stage transition.

**Figure 5:**
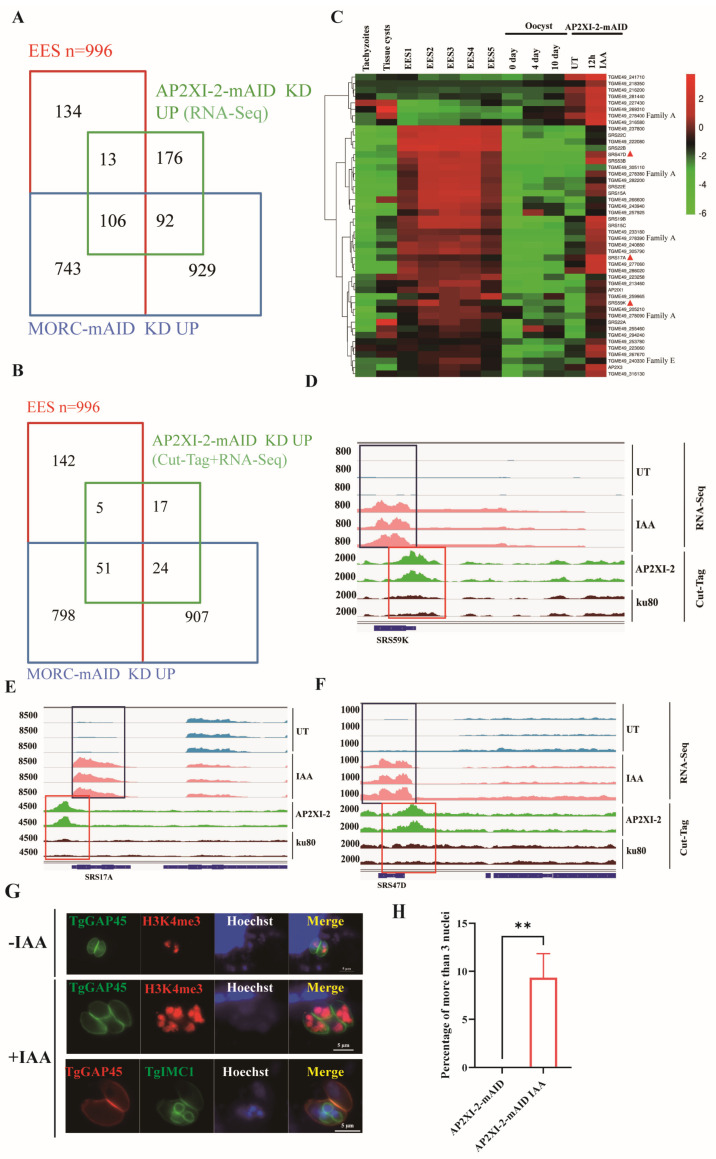
Depletion of AP2XI-2 induces the expression of enteroepithelial-stage-restricted transcripts. (**A**) Venn diagram of genes upregulated after AP2XI-2 knockdown (n = 387 genes) and Cut-Tag peaks (n = 97) (**B**) for AP2XI-2 against EES-specific genes [22] and genes upregulated after MORC [9] knockdown (n = 1870 genes). (**C**) Hierarchical clustered transcriptional heatmap showing genes highly upregulated (Log2FC ≥ 2, RNA-seq) after AP2XI-2 depletion and AP2XI-2-bounded targets (Cut-Tag data). The transcript abundance at various developmental stages, namely the tachyzoite, bradyzoite/cyst, EES, and oocyst stages, is displayed. Triangles represent genes of interest. The red triangle represents the genes we’re interested in. (**D**–**F**) IGV screenshots of the genomic regions of the three merozoite-specific SRS genes. Cut-Tag profiles were obtained using antibodies directed against HA (AP2XI-2 tagged) in chromatin sampled from the AP2XI-2-mAID-HA strain and the Δku80 strain. RNA-seq data from AP2XI-2-mAID-HA after 12 h of treatment or no treatment with IAA are shown in red and blue peaks. The normalized RPKMs for Cut-Tag and RNA-seq reads are shown on the *y*-axis. (**G**) IFA showing the patterns of daughter cells budding (as revealed by IMC1 staining) in the AP2XI-2-mAID strain treated with IAA, as determined in Figure 4I. (**H**) Statistics of multi-nucleus parasites (n ≥ 3) that formed after AP2XI-2 knockdown. Parasites were treated with IAA for 12 h. The number of nuclei was determined by three independent assays of 100 parasites each. **, *p* < 0.005.

Although the downstream genes of AP2XI-2 and MORC showed only a statistically low correlation (Figure 3F), we found that the target genes of AP2XI-2 exhibited a significant EES correlation. Upon AP2XI-2 knockdown, 119 of the 387 upregulated genes (30.7%) were EES-specific, of which 106 overlapped with MORC-regulated EES genes (Figure 5A, Table S5). This was also discovered by Antunes et al. [15], who found that 154 of the 573 genes regulated by AP2XI-2 were highly expressed in the EES stage (Figure S12A–C). Combined with the Cut-Tag data on the AP2XI-2 protein, we found that the promoters of 97 of the 387 upregulated genes were able to be bound and regulated by the AP2XI-2 protein (Figure 5B, Table S6). The majority of these genes were also specific to the EES stage, including merozoite markers such as GRA11b [28] and the four merozoite-restricted *T. gondii* family A proteins [22] (Figure 5C). In addition, AP2XI-2 regulates a substantial number of SRS surface proteins (Table S7). Upon loss of AP2XI-2, many SRS surface proteins showed a high degree of upregulation (Log2FC ≥ 2), 85% (17/20) of which were EES-stage-specific proteins [23] (Figure S13A). The expression trend of these SRS surface proteins is consistent with the expression pattern observed after MORC depletion [9] (Figure S13B). Meanwhile, the promoter regions of these SRS genes (e.g., SRS59K, SRS17A, and SRS47D) were also observed to bind to AP2XI-2 (Figure 5D–F), resulting in transcriptional inhibition.

As the target genes of AP2XI-2 are correlated to MORC targets and EES genes, the endopolygeny division was also tested in the AP2XI-2-deficient parasites. After 12 h of IAA treatment, about 10% of the AP2XI-2-mAID parasites showed characteristics of endopolygeny with multiple (more than two) nuclei and daughter cells within an individual mother cell (Figure 5G,H). Wang et al.’s results also support this phenomenon [17]. In summary, these results indicate that both AP2XI-2 and AP2XII-1 exhibit characteristics that inhibit the expression of merozoite-specific genes in *T. gondii* and are key to the parasite stage transition.

### 2.5. AP2XII-1 and AP2XI-2 Co-Regulate Specific Genes in Enteroepithelial Stages

**Figure 6:**
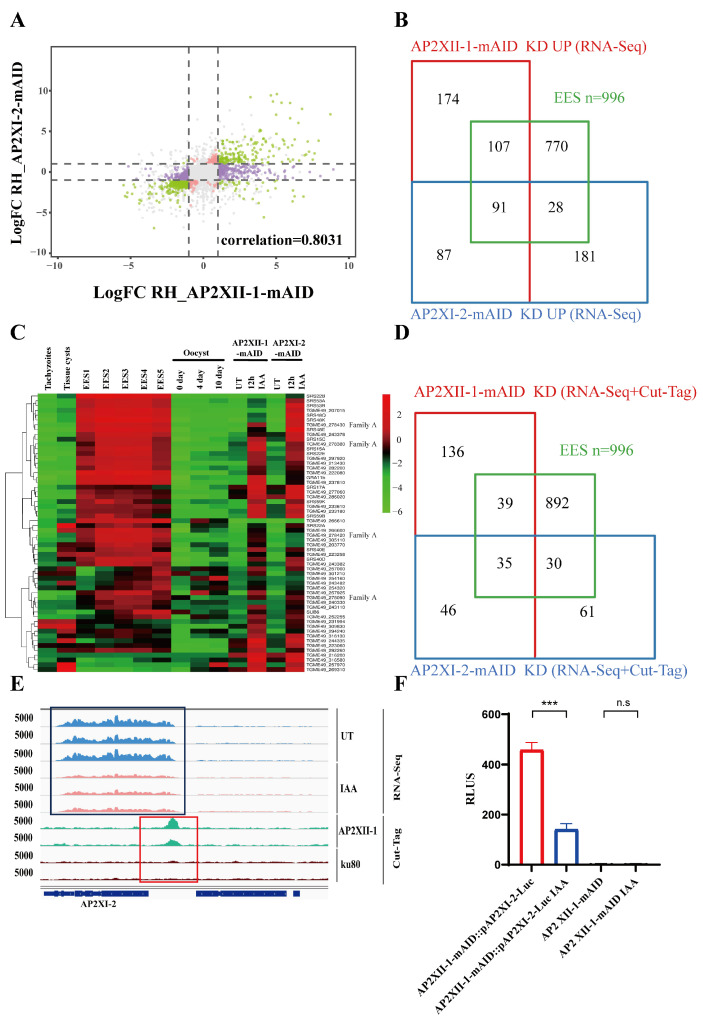
Highly correlated downstream genes regulated by AP2XII-1 and AP2XI-2. (**A**) Pearson correlation between gene alterations after knockdown of AP2XII-1 and AP2XI-2. Green dots represent positive correlated DEGs after AP2XII-1 and AP2XI-2 knockout. (**B**) Venn diagram of genes upregulated after AP2XI-2 (n = 387 genes) or AP2XII-1 (n = 459 genes) knockdown against the EES-specific genes [22]. (**C**) Hierarchical clustered transcriptional heatmap showing highly upregulated genes (Log2FC ≥ 2, RNA-seq) after AP2XI-2 or AP2XII-1 knockdown. The transcript abundance at various developmental stages, namely the tachyzoite, bradyzoite/cyst, EES, and oocyst stages [24], is displayed. (**D**) Venn diagram of Cut-Tag hits and upregulated genes after AP2XI-2 (n = 172 genes) or AP2XII-1 (n = 256 genes) knockdown against the EES-specific genes. (**E**) IGV screenshots of the genomic region of AP2XI-2. Cut-Tag profiles were obtained using antibodies directed against HA (AP2XII-1-tagged) in chromatin sampled from the AP2XII-1-mAID-HA strain and the Δku80 strain. RNA-seq data from AP2XII-1-mAID-HA parasites after 12 h of treatment or no treatment with IAA are shown in red and blue peaks. The normalized RPKMs for Cut-Tag and RNA-seq reads are shown on the *y*-axis. (**F**) Relative RLUs of AP2XII-1-mAID-HA parasites expressing luciferase under the control of the AP2XI-2 promoter. pAP2XI-2-Luc::AP2XII-1-mAID and AP2XII-1-mAID were treated with IAA or a vehicle for 12 h and then detected for luciferase expression. ***, *p* < 0.001; n.s, no significant difference.

Since both AP2XI-2 and AP2XII-1 regulate *T. gondii* genes during the EES stages, we further explored the relationship between AP2XI-2 and AP2XII-1. Based on RNA-seq data analysis, the Pearson correlation coefficient between the gene sets regulated by AP2XI-1 and AP2XI-2 was 0.8031, indicating a strong correlation (Figure 6A). The Venn diagram showed that 178 (178/459, 38.8%) upregulated genes in the AP2XII-1 knockdown parasite overlapped with the upregulated genes after AP2XI-2 knockdown (178/387, 46.0%) (Figure 6B). Of the 178 overlapping genes, 91 genes (91/178, 51.1%) were EES-specific, 15 (15/178, 8.4%) genes were bradyzoite-stage-specific [21], and 6 (6/178, 3.3%) genes were sporozoite-stage-specific [24]. Notably, among the 58 genes (Log2FC ≥ 2) that were the most significantly upregulated in AP2XII-1- and AP2XI-2-deficient parasites, 68% (40/58) were EES-specific genes, including GRA11b and 13 SRS surface antigens [22]. In addition, four specific *T. gondii* A family proteins were also co-regulated by AP2XII-1 and AP2XI-2 (Figure 6C) [22]. These findings provide evidence of the co-regulation of the AP2XII-1 and AP2XI-2 genes during the EES stage of *T. gondii*.

Combined with the Cut-Tag data, we found that 256 gene promoters were bound by AP2XII-1 out of 1197 differentially expressed genes in the AP2XII-1 knockdown parasite. Of these 256 gene promoters, 32% (81/256) of them were also bound by AP2XI-2. Similarly, of the 172 gene promoters that were bound by AP2XI-2, 47% (81/172) were also bound by AP2XII-1 (Figure 6D). Notably, 35 EES-specific genes were identified among the 81 overlapping genes co-regulated by AP2XI-2 and AP2XII-1, including GRA11b [28], five SRS surface proteins (SRS15A, SRS17A, SRS22E, SRS59K, and SRS19B) and four *T. gondii* A family proteins [22]. These results confirm the strong correlation between AP2XII-1 and AP2XI-2, which collectively regulate EES-stage-specific gene sets.

More interestingly, a significant decrease (Log2FC = −1.29, *q*-value = 1.87 × 10^−65^) in the transcription level of AP2XI-2 was found in the RNA-seq results of the AP2XII-1-deficient parasite (Figure 6E). Moreover, the Cut-Tag results indicate that AP2XII-1 could bind to the AP2XI-2 promoter (Figure 6E). To demonstrate the regulatory effect of AP2XII-1 on the AP2XI-2 promoter, a luciferase reporter system driven by the AP2XI-2 promoter based on AP2XII-1-mAID parasites was constructed (Figure S11A). After 12 h of IAA treatment, the luciferase expression in AP2XII-1-mAID::pAP2XI-2-Luc parasites was significantly decreased compared with untreated parasites (Figure 6F). These results indicate that, in addition to co-repressing the downstream EES gene sets, AP2XII-1 can also provide positive feedback to AP2XI-2.

### 2.6. AP2XII-1 Is a Primary AP2 and Regulates the Expression of Multiple AP2 Genes

**Figure 7:**
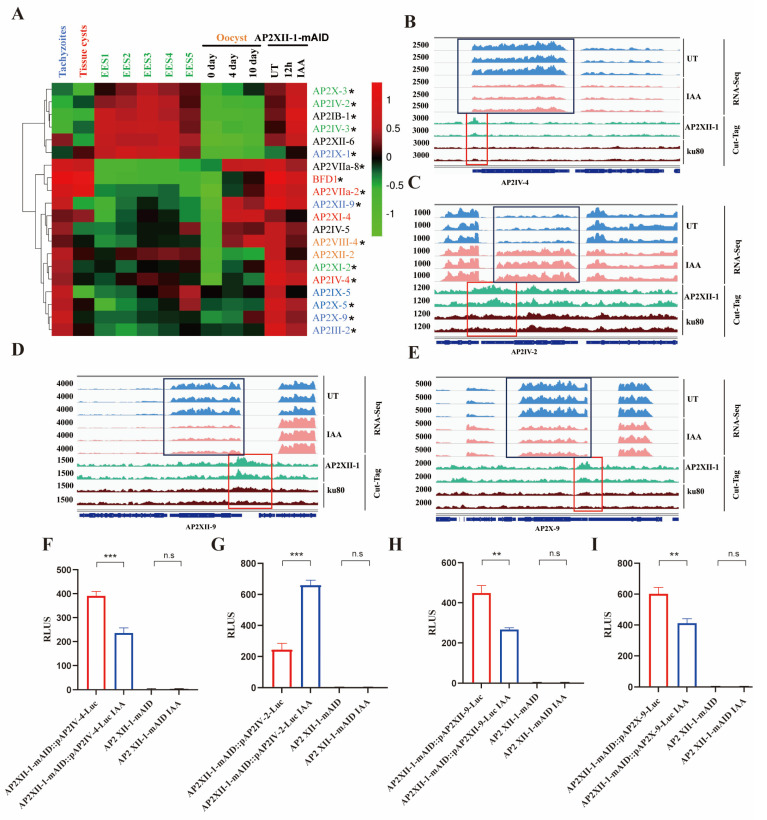
AP2XII-1 is a primary AP2 that regulates the transcription of downstream secondary transcription factors. (**A**) Hierarchical clustered transcriptional heatmap showing transcriptional factor differences after AP2XII-1 depletion. The abundance of transcripts in various developmental stages, namely the tachyzoite (blue), bradyzoite (red), EES (green), and oocyst (yellow) stages, is shown. Asterisks represent AP2XII-1 bound to its promoter (Cut-Tag). (**B**–**E**) IGV screenshots of the genomic regions of the four AP2 genes. Cut-Tag profiles were obtained using antibodies directed against HA (AP2XII-1-tagged) in chromatin sampled from the AP2XII-1-mAID-HA strain and the Δku80 strain. RNA-seq data from AP2XII-1-mAID-HA parasites after 12 h of treatment or no treatment with IAA are shown in red and blue peaks. The normalized RPKMs for Cut-Tag and RNA-seq reads are shown on the *y*-axis. (**F**–**I**) Relative RLUs of AP2XII-1-mAID-HA parasites expressing luciferase under the control of the AP2 promoters. These AP2-promoter-driven luciferase-expressing AP2XII-1-mAID-HA parasites and the parental AP2XII-1-mAID-HA strain were treated with IAA or a vehicle for 12 h and then detected for each treatment for luciferase expression. **, *p* < 0.01; ***, *p* < 0.001; n.s, no significant difference.

Apart from AP2XI-2, we also found that 19 transcription factors, including 18 ApiAP2s and BFD1, were differentially expressed after AP2XII-1 knockdown (Figure 7A). Multiple merozoite-stage-specific ApiAP2s (AP2IV-2 [9], AP2IV-3 [29], and AP2X-3 [9]) were significantly upregulated in AP2XII-1-deficient strains. This further supports our previous results indicating the gene transcription switch of *T. gondii* tachyzoites to the merozoite stage after AP2XII-1 knockdown.

Notably, AP2XII-1 deficiency inhibits the transcription of AP2XI4, AP2IV4, and BFD1, which has been shown to be associated with the differentiation of tachyzoites into bradyzoites in vitro as well as the formation of cysts in animals [30,31]. Specifically, BFD1 has been proven to be a master regulator of bradyzoite differentiation. In addition, loss of AP2XII-1 results in the inhibition of six ApiAP2 genes (AP2IX-1, AP2III-2, AP2X-9, AP2X-5, AP2XII-9, and AP2IX-5) that regulate the tachyzoite-stage gene set (Figure 7A, blue). AP2X-5 is crucial for the expression of S/M-phase virulence genes [32]. AP2IX-1 is a transcription factor that controls the switch from the ubiquitous SAG1 to rare surface antigens in tachyzoites [33]. AP2IX-5 has been shown to control the activation of the S/M-specific cell cycle expression program in tachyzoites, while inhibition of AP2IX-5 expression permits a switch from endodyogeny to endopolygeny division patterns [20,34]. These results indicate that AP2XII-1 deficiency inhibits the expression of gene sets in both tachyzoite and bradyzoite stages, thereby inhibiting the transformation of parasites into tachyzoites and bradyzoites.

Cut-Tag results show that AP2XII-1 binds to the promoters of AP2IB-1, AP2IV-2, AP2IV-3, AP2IX-1, AP2X-3, AP2VIIa-2, AP2VIII-4, AP2VIIa-8, AP2XI-2, AP2X-9, AP2X-5, AP2XII-9, AP2IV-4, AP2III-2, and BFD1 (Table S8). To demonstrate the binding and regulation of these ApiAP2 promoters by AP2XII-1, we tested the promoters of AP2XI-2, AP2IV-2, AP2IV-4, AP2X-9, and AP2XII-9 by our luciferase reporter system in AP2XII-1-mAID parasites. The results show that, upon loss of AP2XII-1, the expression trends of these ApiAP2-promoter-regulated luciferase enzymes were altered as shown in the transcriptomic data (Figure 6E,F and Figure 7B–I, Table S8).

Interestingly, based on RNA-seq and Cut-Tag data, we observed that five of the seven ApiAP2s regulated by AP2XI-2 were also regulated by AP2XII-1 (Figure S14A). Meanwhile, the regulatory trends of AP2XII-1 and AP2XI-2 on these five AP2s are completely consistent (Figure S14B). AP2XII-9, AP2III-2, and AP2IV-4 were downregulated and AP2X-3 and AP2IX-1 were upregulated when AP2XII-1 or AP2XI-2 was knocked down.

Taken together, these results indicate that AP2XII-1 acts as a primary ApiAP2 that regulates the transcription of multiple downstream ApiAP2s (secondary ApiAP2s), which is consistent with the theoretical model previously proposed by Farhat et al. [9,35].

## 3. Discussion

*T. gondii* employs a complex regulatory network to control its gene expression in response to its diverse and challenging life cycle. Understanding the mechanisms underlying gene regulation is crucial to unraveling the parasite’s pathogenesis and developing effective interventions. Farhat et al. discovered that the microrchidia (MORC) protein in *T. gondii* acts as an upstream transcriptional repressor of sexual commitment. MORC forms a complex with ApiAP2 transcription factors and recruits the histone deacetylase HDAC3, which inhibits the chromatin accessibility of genes specifically expressed during sexual stages [9]. AP2XII-1 and AP2XI-2 are two important transcription factors identified by MORC immunoprecipitation [9]. In this study, AP2XII-1 and AP2XI-2 were found to be crucial for the in vitro growth of tachyzoites. Interestingly, knockdown of AP2XII-1 or AP2XI-2 was found to mainly induce the transition of the parasite from the acute tachyzoite stage to the presexual merozoite stage and upregulate the expression of *T. gondii* enteroepithelial stage (including merozoite and sexual stage)-specific genes, including merozoite markers such as GRA11b [28]. Similarly, a large number of *T. gondii* Family A proteins, thought to be merozoite-specific proteins [22], were significantly upregulated in AP2XII-1-deficient parasites. These results suggest that AP2XII-1 mainly regulates merozoite- and sexual-stage-specific genes, while there is no clear trend toward regulation of bradyzoite-specific genes.

Previous studies have shown that MORC knockdown inhibits the transcription of over 80% of EES-specific genes and also serves as a transcriptional inhibitor of bradyzoite-specific genes (~30% of bradyzoite-specific genes are targeted) [9]. MORC is considered to be a linker between functional HDAC3 and different DNA binding elements (ApiAP2s) that target the promoters of different downstream genes [35]. Thus, these HDAC3–MORC–ApiAP2 complexes subsequently inhibit specific genes at different stages, ultimately controlling the differentiation of *T. gondii*. However, the specific gene cluster regulated by the complexes formed by MORC/HDAC3 with individual ApiAP2s has not been clarified, which limits our understanding of the possible regulatory network [9,35]. Our results indicate that AP2XII-1 and AP2XI-2 maintain a continuous inhibitory state on a wide range of merozoites and sex-specific genes, with significant overlap with the target genes of MORC/HDAC3. Recently, Antunes et al. [15] confirmed the direct binding of AP2XII-1 and AP2XI-2 as well as MORC/HDAC3, and their existing results also show that knockdown of AP2XII-1 and AP2XI-2 produces merozoite-specific gene expression and the formation of merozoites. Together, these results unveil the specific working mechanism of the HDAC3–MORC–AP2XII-1–AP2XI-2 complex in controlling the fate of *T. gondii* parasites.

Each developmental stage of *T. gondii* has its own transcription characteristics, and AP2 is believed to regulate specific genes at different developmental stages of the parasite. A total of 68 ApiAP2s have been identified in *T. gondii* and only a few have been functionally characterized [35,36]. Our study shows that AP2XII-1 can regulate the transcription of 19 other ApiAP2 genes and the BFD1 transcription factor. Notably, multiple merozoite-stage-specific ApiAP2s, including AP2IV3, were significantly upregulated in AP2XII-1-deficient strains. This suggests a cascading regulatory network of AP2s involving primary ApiAP2s (including AP2XII-1 and AP2XI-2) and downstream secondary ApiAP2s [9,35].

Among the 68 ApiAP2s found in *T. gondii*, 24 are recognized to be cell-cycle-regulated ApiAP2s [37]. AP2X-5 has been proven to indirectly regulate the promoter of virulence genes expressed in the S/M phase, mainly in synergy with AP2XI-5 [32]. AP2IX-5 has been reported to control the activation of the S/M-specific cell cycle expression program in tachyzoites, whereas inhibition of AP2IX-5 permits a switch from endodyogeny to endopolygeny division patterns [20,34]. Our study showed that AP2XII-1 knockdown inhibited the expression of AP2X-5 and AP2IX-5, suggesting that AP2XII-1 may inhibit the transition from *T. gondii* tachyzoites to merozoites by maintaining the endodyogeny of tachyzoites through AP2X-5 and AP2IX-5.

Several transcription factors have been shown to be involved in the regulation of bradyzoite development in *T. gondii* [30,38]. Deletions of AP2XI-4 inhibit the expression of genes specific to the bradyzoite stage of *T. gondii* and prevent the formation of cysts in animals [31]. The loss of AP2IV-4 cyst wall and bradyzoite surface proteins prevents tissue cyst formation in vivo [31]. BFD1 has been proven to be a switch that regulates the development of tachyzoites and bradyzoites. Loss of BFD1 inhibits the in vitro differentiation of tachyzoites into bradyzoites and the formation of cysts in animals [30]. This study showed that AP2XII-1 knockdown inhibits the transcription of several transcription factors associated with the development of bradyzoites, such as AP2XI-4, AP2IV-4, and BFD1. Therefore, we speculate that AP2XII-1 may play a role in inhibiting the transition of *T. gondii* to the bradyzoite stage. Paradoxically, we found that the transcription level of AP2IV-3 was significantly upregulated in AP2XII-1-deficient parasites, whereas AP2IX-9 was unaffected. Previous studies have shown that AP2IX-9 shares the same set of target genes as AP2IV-3 [29]. The absence of AP2IV-3 reduces the formation of tissue cysts, whereas the absence of AP2IX-9 leads to an increase in cyst formation [27,29]. Thus, this is in contrast to the regulatory results on AP2XI4, AP2IV4, and BFD1. Recently, Fan et al. demonstrated that depletion of AP2XII-1 causes bradyzoites to spontaneously transition in the *T. gondii* ME49 strain [16].

Notably, Antunes et al. [15] showed that AP2XII-1 and AP2XI-2 form complexes with MORC/HDAC3 to regulate downstream genes. Our study shows that AP2XII-1 can bind to the promoter and regulate the gene expression of AP2XI-2. Moreover, the five AP2s (AP2X-3, AP2XII-9, AP2IX-5, AP2IV-4, AP2XII-2, AP2IX-1, and AP2III-2) co-regulated by AP2XI-2 and AP2XII-1 showed a consistent regulatory trend when knocking down AP2XI-2 or AP2XII-1. Therefore, AP2XII-1 can not only form a complex with AP2XI-2 and MORC/HDAC3 to regulate downstream merozoite-specific gene sets via ApiAP2 cascades but also provide positive feedback to AP2XI-2. However, the specific regulatory mechanism remains unclear and needs to be further investigated.

## 4. Materials and Methods

### 4.1. Culture of Cells and Parasites

The *T. gondii* RH ΔKu80 strain and derived strains were cultured in vitro by serial passages in human foreskin fibroblasts (HFFs; ATCC, Manassas, VA, USA) or African green monkey kidney cells (Vero cells, a gift from Professor Liu Qun at the Chinese Agricultural University) using Dulbecco’s Modified Eagle’s Medium (DMEM, Macgene, Beijing, China) supplemented with 2% FBS at 37 °C and 5% CO_2_. Cells were maintained in DMEM supplemented with 10% heat-inactivated fetal bovine serum (TransGen Biotech, Beijing, China) and incubated at 37 °C in a 5% CO_2_ environment.

### 4.2. Generation of Transgenic T. gondii Strains

The EuPaGDT Library in the ToxoDB database was used to design the gRNAs in the corresponding gene-specific CRISPR–Cas9 plasmids. The construction of CRISPR–Cas9 plasmids was performed as described previously [39]. Briefly, upstream and downstream Cas9 fragments containing gRNA sequences were amplified and ligated using a seamless cloning kit (Vazyme Biotech, Co., Ltd., Nanjing, China). All primers used in this study are listed in Table S9. AP2XII-1-mAID was constructed by fusing the mAID sequence and a 3 × HA epitope tag to the C-terminus of AP2XII-1. The chloramphenicol resistance gene (CmR) and the TIR1-3Flag expression cassette were amplified and linked to the plasmid (mAID-3HA-CAT-TIR1-3Flag). For the C-terminal epitope tagging of AP2XII-1, 59 bp PCR primers containing 39 bp fragments upstream of the AP2XII-1 translation stop codon and downstream of the gRNA site were designed for PCR amplification from the mAID-3HA-CAT-TIR1-3Flag plasmid. The homologous recombinant PCR fragments were co-transfected with the corresponding CRISPR–Cas9 plasmid in RH ΔKu80 parasites and screened with chloramphenicol. Monoclonal parasites were identified by PCR and IFA. The parasites of AP2XI-2-mAID were constructed in agreement with AP2XII-1-mAID. IAA at a final concentration of 500 μM was used to induce AP2XII-1 and AP2XI-2 degradation when needed [40].

A luciferase reporter gene system was constructed to verify the regulation of gene promoters. To construct RH Δku80::pSAG1-Luc parasites, a firefly luciferase gene with the TgSAG1 promoter was inserted at the UPRT locus of the RH Δku80 strain. The pGRA11b-Luc plasmid was constructed by replacing the TgSAG promoter with the TgGRA11b promoter. The plasmid was transfected into the AP2XII-1-mAID strain to construct pGRA11b-Luc::AP2XII-1-mAID parasites. The strategy for the other promoters validated in this study is consistent with pGRA11b.

### 4.3. Intracellular Replication Assay and Invasion Assay

An intracellular replication assay was performed to determine the number of parasites per vacuole at 24 h post-invasion. HFFs growing in 12-well plates seeded on coverslips were inoculated with 1 × 10^5^ parasites. After 1 h, uninvaded parasites were removed and cultured for 24 h with IAA (500 μM) or a vehicle (Ethyl Alcohol, 1:1000). The parasites were then fixed and co-stained with mouse anti-HA (diluted to 1:500, Sigma, St. Louis, MO, USA) and rabbit anti-αGAP45 polyclonal antibodies (diluted to 1:300, a gift from Professor Qun Liu at the Chinese Agricultural University). Nuclear DNA was stained with Hoechst 33258 (diluted to 1:100, Macgene, Beijing, China). Parasites of each strain in vacuoles were quantified by counting at least 100 vacuoles using a fluorescence microscope (Zeiss, Oberkochen, Germany). Three independent experiments were performed. For the invasion assay, the percentage of invasion was calculated based on the number of vacuoles per host cell. Three independent experiments were conducted.

### 4.4. Plaque Assay

Plaque assays were performed as described previously [41]. To assess the impact of AP2XII-1 and AP2XI-2 on plaque formation, 100 parasites per well were inoculated onto HFF monolayers in 12-well plates and treated with either a vehicle (EtOH, 1:1000) or IAA (500 μM). Infected cells were cultured for 7 days at 37 °C under 5% CO_2_ conditions. Subsequently, infected cells were fixed in 4% PFA for 30 min and stained with crystal violet for 1 h. The plaque area was counted in pixels using Photoshop C6S software (Adobe, USA), with data compiled from three independent experiments.

### 4.5. Immunoblotting and Immunofluorescence Assays

For immunoblotting assays, parasites were collected and purified by filtration through a 5 µm filter membrane and lysed with RIPA buffer (Huaxinbio, Beijing, China). The primary antibodies used were mouse anti-HA (diluted to 1:5000, Sigma) and mouse anti-actin (diluted to 1:6000, Abmart, Shanghai, China). The secondary antibodies used were goat anti-mouse or rabbit (1:5000, Huaxinbio, Beijing, China).

Immunofluorescence assays (IFAs) were used for subcellular localization. Freshly harvested parasites were inoculated onto HFF monolayers grown on glass coverslips in 12-well plates. At 24 h post-infection, infected cells were fixed with 4% PFA for 30 min and permeabilized with 0.25% Triton X-100. The samples were then blocked with 3% bovine serum albumin (BSA) for 30 min. Primary mouse anti-HA (diluted to 1:500, Sigma, USA) antibody, mouse anti-Ty antibody (diluted to 1:300, a gift from Professor Shaojun Long at the Chinese Agricultural University), mouse anti-GRA11b antibody (diluted to 1:500, a gift from Professor Bang Shen’s lab at the Huazhong Agricultural University), rabbit anti-H3k4me9 (1:300, Abmart, Shanghai, China), rabbit anti-TgGAP45 polyclonal antibody (diluted to 1:300), and rabbit anti-TgIMC1 polyclonal antibody (diluted to 1:300, an inner membrane complex marker) were incubated for 1 h. FITC-conjugated goat anti-rabbit IgG (diluted to 1:50, Proteintech, Rosemont, IL, USA) and cy3-conjugated goat anti-mouse IgG (diluted to 1:100, Proteintech, USA) were used as secondary antibodies. Infected monolayers were observed by a fluorescence microscope (Zeiss, Germany).

### 4.6. RNA-Seq and Data Analysis

Transgenic parasite strains cultured in Vero cells were treated with either 500 μM IAA or a vehicle for 12 h. Total RNAs from *T. gondii* tachyzoites were then extracted using the M5 Total RNA Extraction Reagent (Mei5 Biotechnology Co., Ltd., Beijing, China) according to the manufacturer’s protocol. Each treatment consisted of three biological replicates. The purity, concentration, and integrity of RNAs were tested using the NanoPhotometer^®^ (IMPLEN, Westlake Village, CA, USA), the Qubit^®^ RNA Assay Kit in the Qubit^®^ 2.0 Fluorometer (Life Technologies, Carlsbad, CA, USA), and the RNA Nano 6000 Assay Kit of the Bioanalyzer 2100 system (Agilent Technologies, Santa Clara, CA, USA), respectively. Only qualified samples were used to prepare the library. Illumina sequencing libraries were generated using the NEBNext^®^ Ultra™ RNA Library Prep Kit for Illumina^®^ (NEB, Ipswich, MA, USA) according to the manufacturer’s recommendations. Sequencing was performed using the Illumina Novaseq 6000 platform (Shanghai Personal Biotechnology Co., Ltd., Shanghai, China) in order to generate 150 bp paired-end reads. The original sequencing data can be found in the Sequence Read Archive database under the accession number PRJNA1009254. Paired-end clean reads were aligned to the *T. gondii* ME49 reference genome (ToxoDB, version 57) using Hisat2 v 2.2.1 [42]. The resulting sam files were transformed into bam files. The read count per gene for each sample was calculated using the sorted bam files by htseq-count v 0.13.5 [43]. Differentially expressed genes were calculated by the DEseq2 R package [44]. Gene ontology enrichment analysis was performed in ToxoDB. Gene expression with a fold change >2 or <−2 and an adjusted *p*-value of <0.01 was defined as significantly differentially expressed. The transcripts per kilobase million (TPM) were calculated for each gene and used to draw clustered heatmaps.

### 4.7. Cut-Tag and Data Analysis

The transgenic strains were inoculated in 25 cm^2^ cell culture flasks and washed after 4 h to remove non-invasive parasites. Transgenic strains (1 × 10^5^) were harvested after 12 h of incubation with or without IAA (500 μM). Library construction was performed using the Hyperactive Universal CUT&Tag Assay Kit for Illumina (Vazyme, Nanjing, China) according to the manufacturer’s instructions. Briefly, fresh tachyzoites were bound to activated concanavalin A beads (10 mL/sample) and incubated for 10 min at room temperature. The mixture was resuspended and incubated with primary antibody (1:50, mice anti-HA) at 4 °C overnight. After several washes, the parasites were incubated with secondary antibody (1:100, goat anti-mouse IgG) for 1 h at room temperature. The parasites were then resuspended with 300 mL of pA-Tn5 buffer (0.04 mM) and incubated for 1 h at room temperature on a rotator. Tagmentation was stopped by proteinase K treatment and DNA extraction was performed using DNA extraction beads (Vazyme, Nanjing, China) [45]. Illumina sequencing libraries were generated by PCR amplification with specific adaptors according to the manufacturer’s recommendations (TruePrep Index Kit V4 for Illumina, Vazyme, Nanjing, China). Cut-Tag libraries were sequenced using the Illumina Novaseq 6000 platform (Shanghai Personal Biotechnology Co., Ltd.).

The paired-end reads were filtered and then aligned to the *T. gondii* reference genome using Bowtie2 [46] (v.2.1.0). The resulting sam files were transformed into bam files. PCR duplicates were removed from the sorted bam files using Sambamba [47]. The filtered reads were then employed to identify Cut-Tag peaks using MACS2 [48]. The overlapping peaks in two biological replicates were identified by the Irreproducibility Discovery Rate (IDR) [49]. The final peaks were annotated against the latest *T. gondii* data in ToxoDB. The sorted and filtered bam files of Cut-Tag peaks and RNA-seq reads were normalized to the RPKM with a resolution of 10 bp (bin size) and transformed into bigwig files for direct visualization in the Integrative Genomics Viewer (IGV) [50]. All raw sequencing data can be found in the Sequence Read Archive database under the accession number PRJNA1009254.

### 4.8. Luciferase Assays

A total of 1 × 10^6^ transgenic parasites expressing luciferase were inoculated into Vero cells in a 96-well plate. After 12 h, the cells were treated with IAA or a vehicle for 12 h. The cells were then harvested, and the relative luminescence units (RLUs) were detected by a fluorescence microplate reader (Tecan, Infinite M200 PRO, Männedorf, Switzerland) using the Bright-Lumi™ II Firefly Luciferase Assay Kit (Beyotime Biotech, Shanghai, China).

### 4.9. Statistical Analysis

Violin charts, line drawings, scatter plots, and histograms were generated using GraphPad Prism 9 (San Diego, CA, USA). Heatmaps and Venn diagrams were drawn using the OmicStudio tools, which can be found at https://www.omicstudio.cn/tool (accessed on 13 January 2023). All experiments were performed in independent biological replicates as described above for each experiment. Statistical significance in the plaque, invasion, proliferation, and parasite growth inhibition assays was evaluated by two-tailed unpaired *t*-tests or two-way ANOVA using GraphPad Prism. Statistical data are expressed as the mean value ± standard error.

## Data Availability

All datasets generated for this study are included in the manuscript or the supplementary files. All raw Cut-Tag sequencing data can be found in the Sequence Read Archive database under the accession number PRJNA1009254, and the original RNA-Seq data can be found in the Sequence Read Archive database under the accession number PRJNA1009254.

## Supplementary Materials

The following supporting information can be downloaded at: https://www.mdpi.com/article/10.3390/ijms25105527/s1, References [51,52,53,54] are cited in the supplementary materials.
