# Supplementary material for: AP2XII-1 and AP2XI-2 Suppress Schizogony Gene Expression in Toxoplasma gondii
Source: Int J Mol Sci. 2024 May 18;25(10):5527. doi: 10.3390/ijms25105527 (PMC11122372; doi:10.3390/ijms25105527)
Supplement: Supplementary file 1 [file ijms-25-05527-s001.zip › Figure S1.pdf]

A

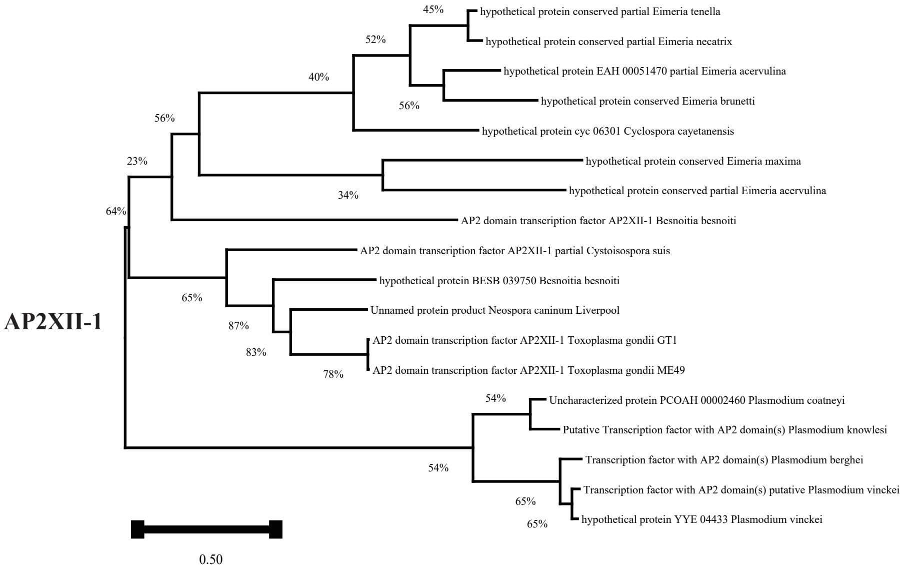

B

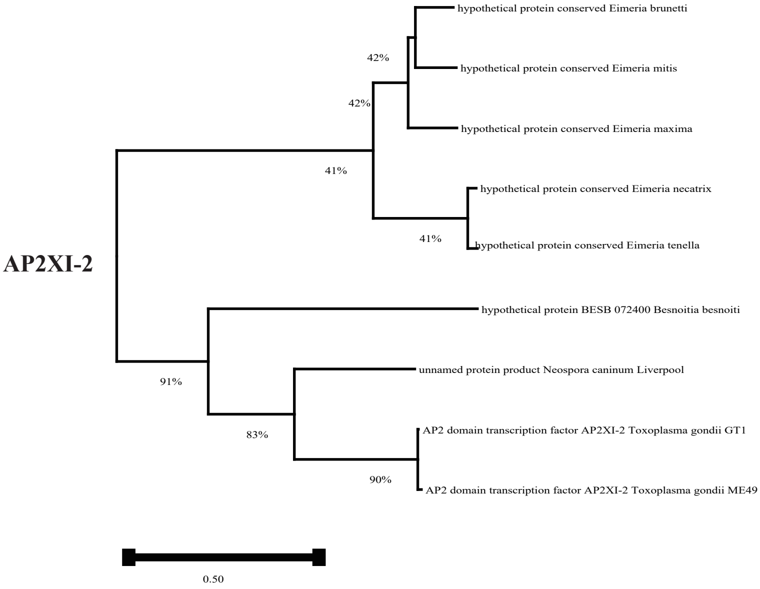

Figure S1. Phylogenetic trees demonstrating the relationship between the Toxoplasma ApiAP2 proteins and homologs sequences in other apicomplexan parasites. The numbers at the nodes indicate the percent out of 1000 bootstrapping trials. The full protein sequences from AP2XII-1 (A), AP2XI-2 (B) were used for blast to find their homologs.
