# Supplementary material for: AP2XII-1 and AP2XI-2 Suppress Schizogony Gene Expression in Toxoplasma gondii
Source: Int J Mol Sci. 2024 May 18;25(10):5527. doi: 10.3390/ijms25105527 (PMC11122372; doi:10.3390/ijms25105527)
Supplement: Supplementary file 1 [file ijms-25-05527-s001.zip › Figure S11.pdf]

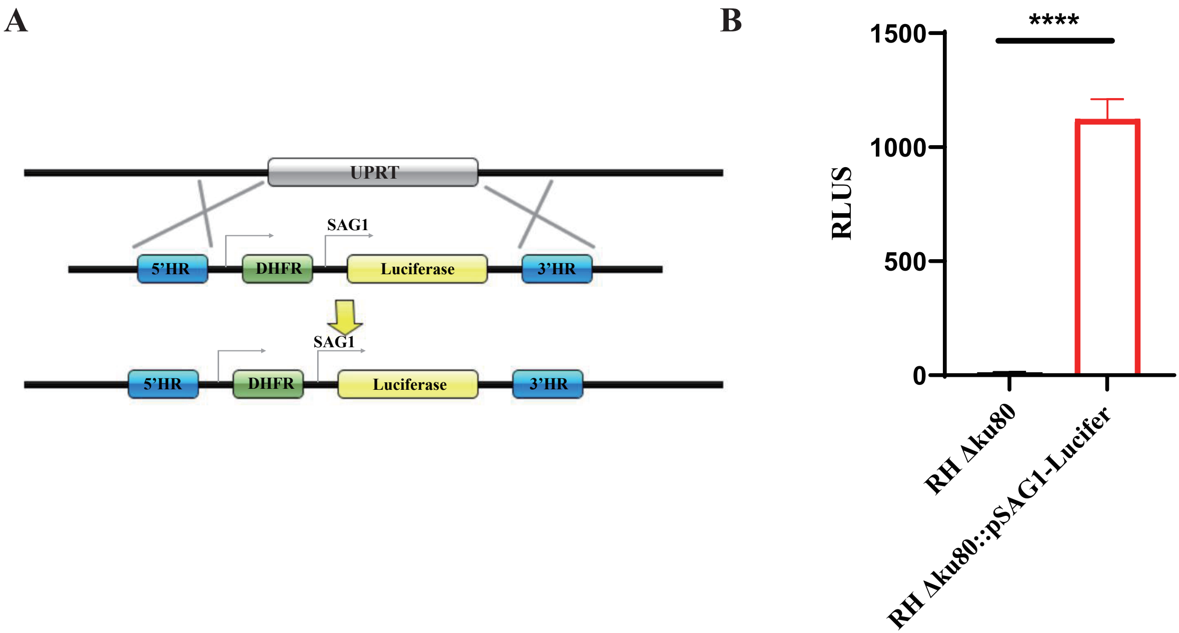

Figure S11. Construction of a luciferase reporter system. (A) Diagram showing the construction strategy for the luciferase reporter parasite. Briefly, genes encoding firefly luciferase driven by the TgSAG1 promoter were inserted into the TgUPRT locus of the RH-Δku80 or AP2XII-1-mAID-HA strains via CRISPR-mediated DSB. To validate the transcriptional activity of promoters, the TgSAG1 promoter was replaced with their promoter sequences and the resulting donor plasmids were used for transcription of RH-Δku80 or AP2XII-1-mAID-HA strains. (B) Identification of the luciferase reporter system. The luciferase-expressing parasite driven by the TgSAG1 promoter (pSAG1-Luc::RH-Δku80) and parental RH-Δku80 were lysed and luciferase expression was determined as relative RLUs. The experiment was repeated independently three times. ****: $P < 0.0001$.
