# Supplementary material for: AP2XII-1 and AP2XI-2 Suppress Schizogony Gene Expression in Toxoplasma gondii
Source: Int J Mol Sci. 2024 May 18;25(10):5527. doi: 10.3390/ijms25105527 (PMC11122372; doi:10.3390/ijms25105527)
Supplement: Supplementary file 1 [file ijms-25-05527-s001.zip › Figure S12.pdf]

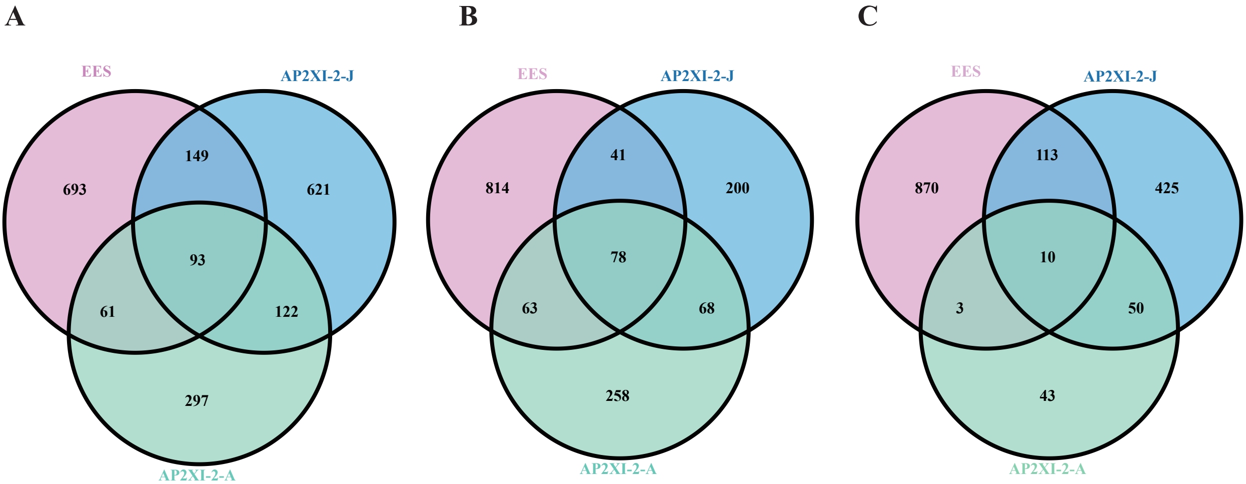

Figure S12. Highly overlap of differently expressed genes after AP2XI-2 knock-down in data from Antunes et al and this study. The RNA-seq data from Antunes et al and this study were compared, and the overall DEGs (A), upregulated genes (B), and downregulated genes (C) were overlapped by Venn diagrams. AP2XI-2-A, and AP2XI-2-J represents DEGs from Antunes et al and this study, respectively.
