# Supplementary material for: AP2XII-1 and AP2XI-2 Suppress Schizogony Gene Expression in Toxoplasma gondii
Source: Int J Mol Sci. 2024 May 18;25(10):5527. doi: 10.3390/ijms25105527 (PMC11122372; doi:10.3390/ijms25105527)
Supplement: Supplementary file 1 [file ijms-25-05527-s001.zip › Figure S13.pdf]

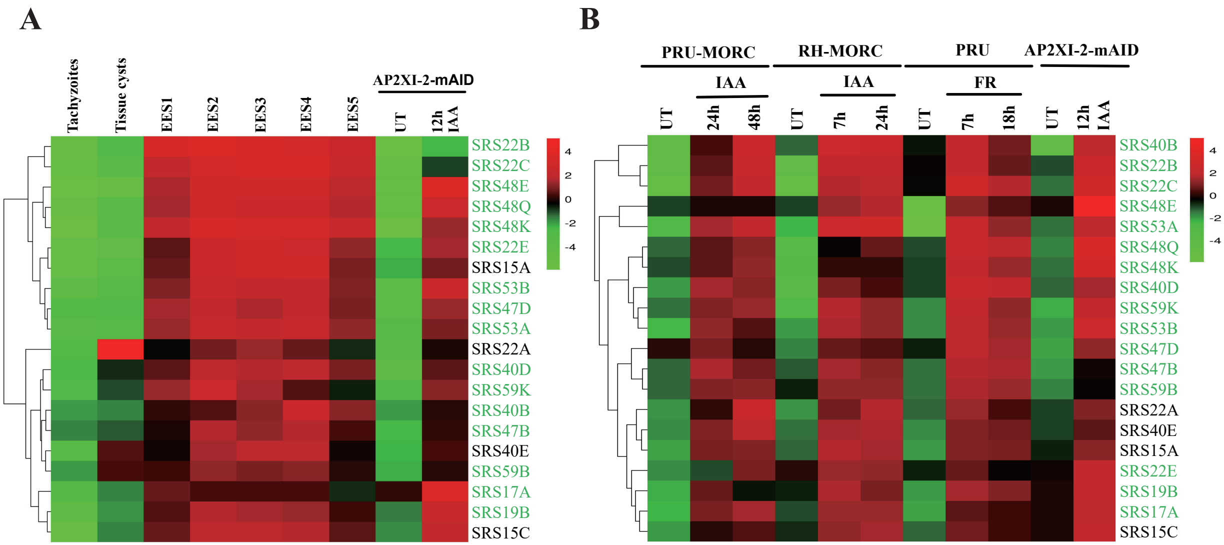

Figure S13. AP2XI-2 knockdown induces expression of EES-specific SRS sur-face proteins. (A) Heatmap showing highly upregulated SRS genes (Log2FC≥2) after AP2XI-2 knockdown, with emphasis on their expression at different life stages. Their transcript abundance at various developmental stages, namely tachyzoite, bradyzoite/cyst and EES stages, is displayed. The names of specific SRS proteins in the EES stage are shown in green font. (B) Heatmap showing highly upregulated SRS genes (Log2FC≥2) after AP2XI-2 and MORC knockdown.
