# Supplementary material for: AP2XII-1 and AP2XI-2 Suppress Schizogony Gene Expression in Toxoplasma gondii
Source: Int J Mol Sci. 2024 May 18;25(10):5527. doi: 10.3390/ijms25105527 (PMC11122372; doi:10.3390/ijms25105527)
Supplement: Supplementary file 1 [file ijms-25-05527-s001.zip › Figure S14.pdf]

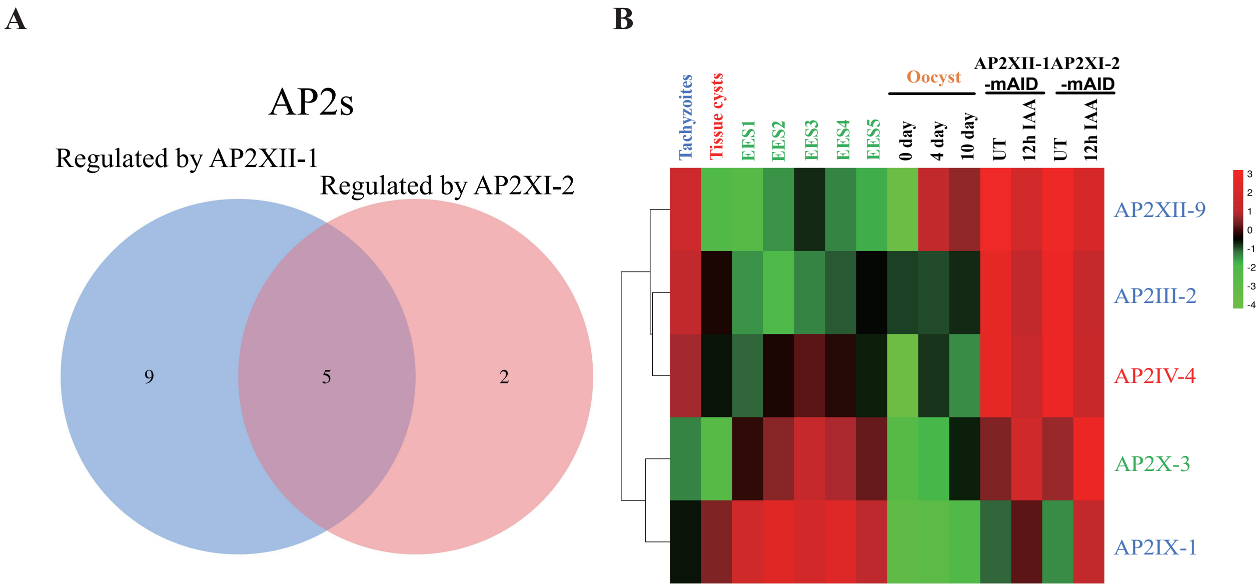

Figure S14. AP2XI-2 and AP2XII-1 jointly bind to the promoters of other ApiAP2s and regulate their transcription. (A) Venn diagram showing the overlap of ApiAP2 proteins with hits in both Cut-Tag peaks and RNA-seq after AP2XII-1 and AP2XI-2 knockdown. (B) Heatmap showing the co-regulated AP2s by AP2XI-2 and AP2XII-1, with emphasis on their expression at different life stages.
