# Supplementary material for: AP2XII-1 and AP2XI-2 Suppress Schizogony Gene Expression in Toxoplasma gondii
Source: Int J Mol Sci. 2024 May 18;25(10):5527. doi: 10.3390/ijms25105527 (PMC11122372; doi:10.3390/ijms25105527)
Supplement: Supplementary file 1 [file ijms-25-05527-s001.zip › Figure S2.pdf]

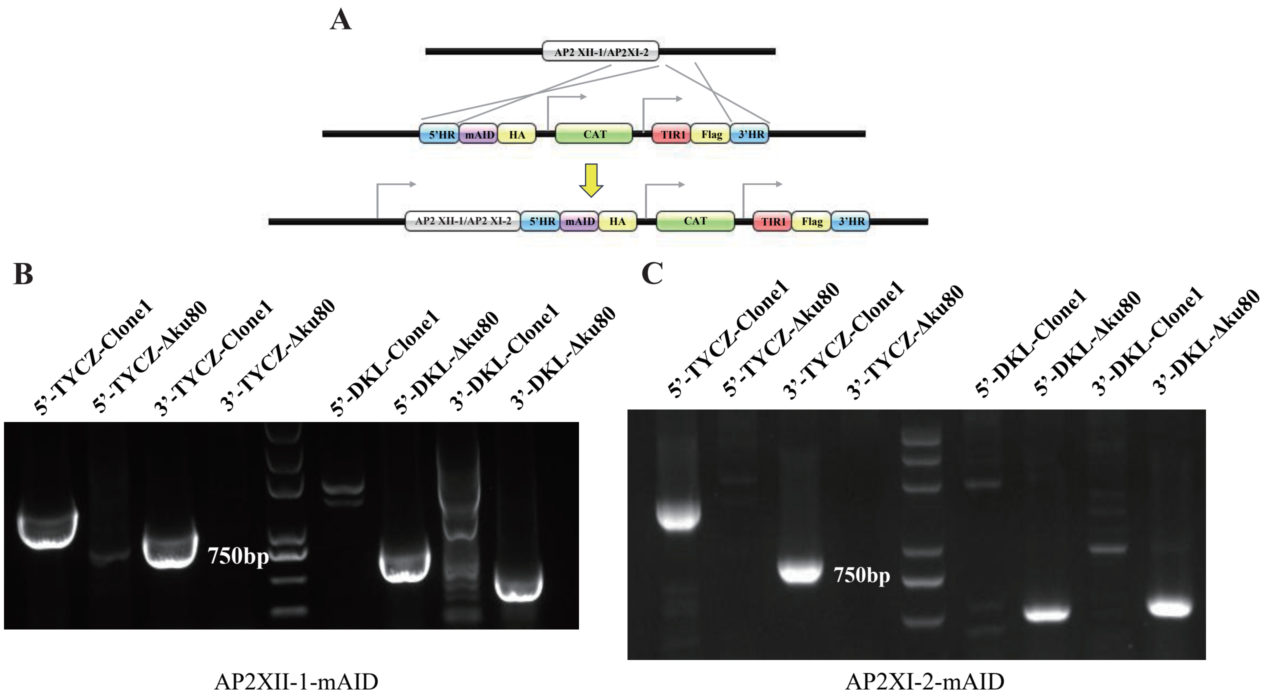

Figure S2. Construction and identification of AP2XII-1 and AP2XI-2 conditional knockdown parasites. (A) Diagram showing the construction strategy of the parasites.
(B-C) Confirmation of recombinant and clonal lines of AP2XII-1-mAID-HA and AP2XI-2-mAID-HA by PCR.
