# Supplementary material for: AP2XII-1 and AP2XI-2 Suppress Schizogony Gene Expression in Toxoplasma gondii
Source: Int J Mol Sci. 2024 May 18;25(10):5527. doi: 10.3390/ijms25105527 (PMC11122372; doi:10.3390/ijms25105527)
Supplement: Supplementary file 1 [file ijms-25-05527-s001.zip › Figure S3.pdf]

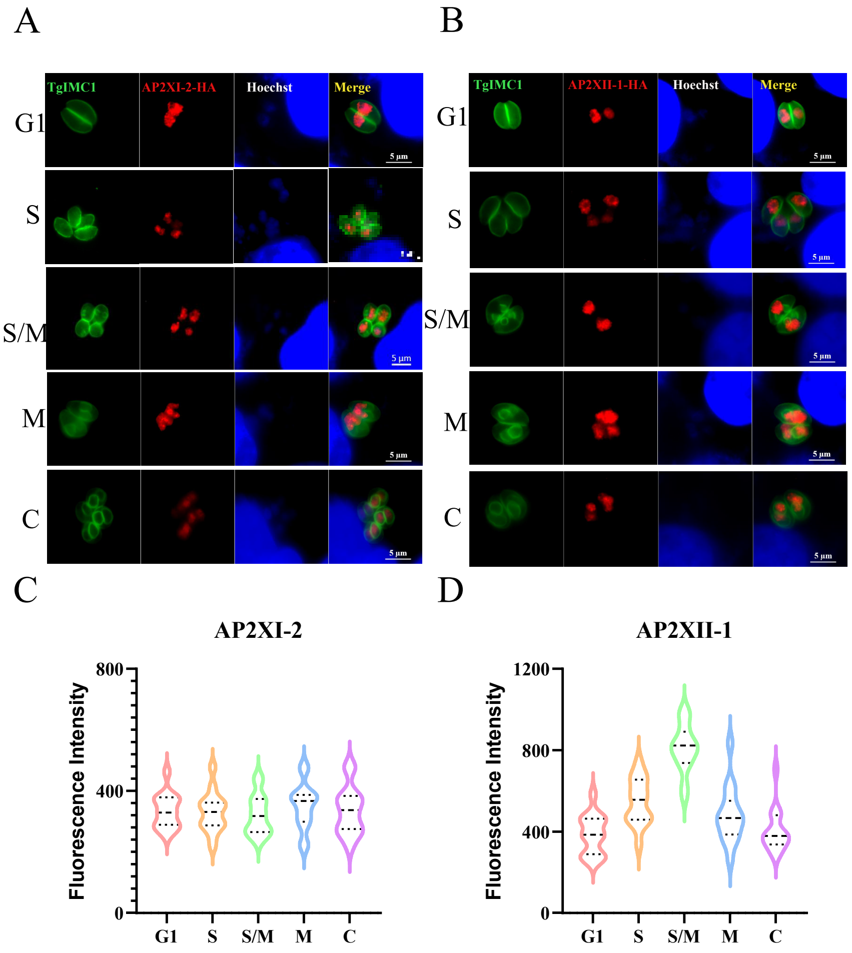

Figure S3. Expression patterns of AP2XII-1 and AP2XI-2 proteins during the tachyzoite cell cycle. Co-staining of AP2XI-2 (A), AP2XII-1 (B) tachyzoites in HFF cells was performed with rabbit anti-inner membrane complex-1 (TgIMC1, green) antibody and mouse anti-HA (red) antibody. Hoechst33258 was used to stain nuclei. IMC1 staining was used to demonstrate tachyzoite cell cycle. Cell cycle stages are shown on the left side of the images. ZEN 3.0 software was used to calculate the mean fluo-rescence intensity of AP2XI-2 (C), AP2XII-1 (D) expressed in different cycles.
