# Supplementary material for: AP2XII-1 and AP2XI-2 Suppress Schizogony Gene Expression in Toxoplasma gondii
Source: Int J Mol Sci. 2024 May 18;25(10):5527. doi: 10.3390/ijms25105527 (PMC11122372; doi:10.3390/ijms25105527)
Supplement: Supplementary file 1 [file ijms-25-05527-s001.zip › Figure S4.pdf]

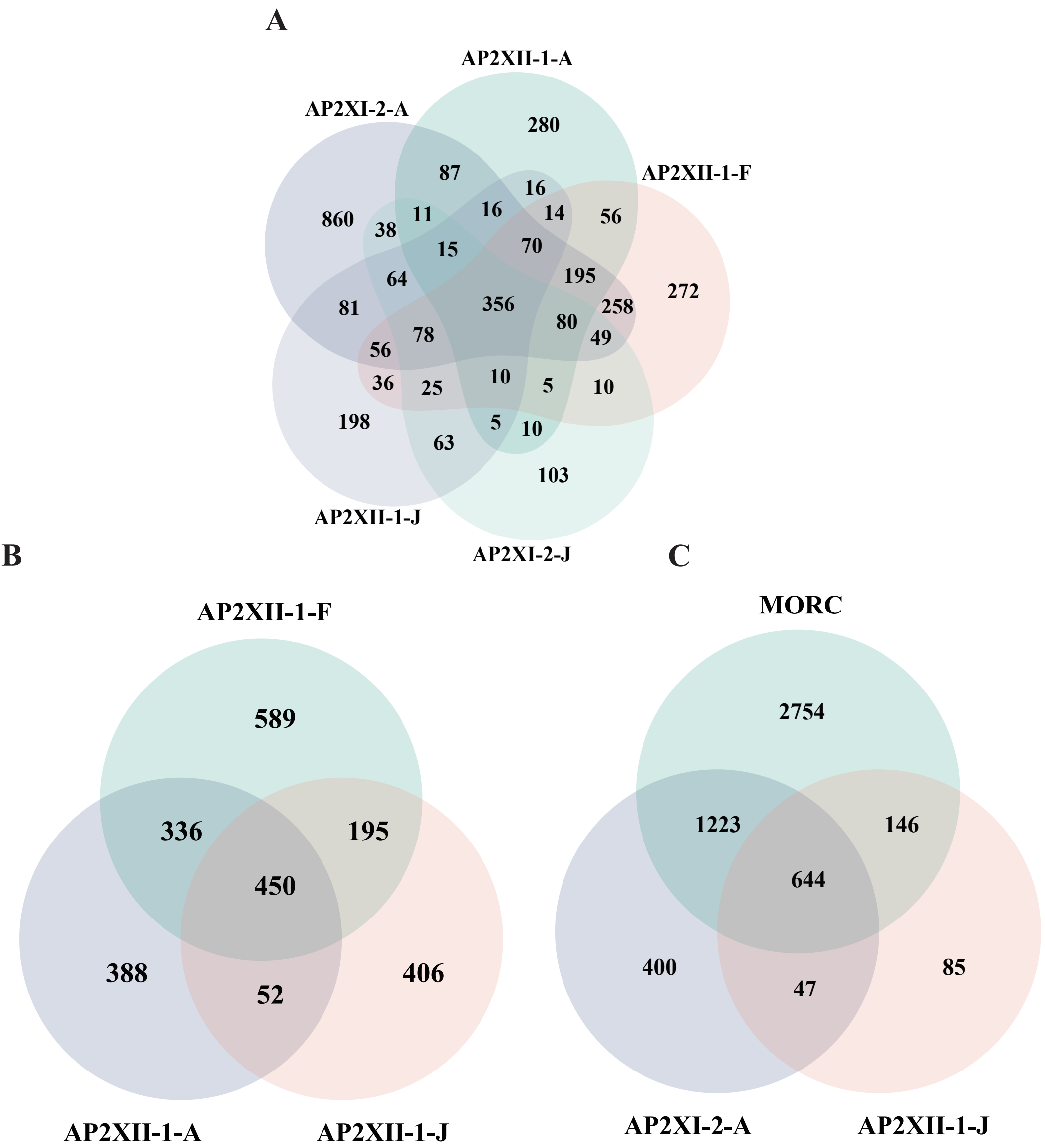

Figure S4. AP2XII-1, AP2XI-2 had good repeatability with downstream promoters regulated by MORC. (A) Venn diagram analysis of peaks for MORC (n=4767), AP2XII-1-J (n=1103), AP2XII-1-F (n=1570), AP2XII-1-A (n=1226), AP2XI-2-J (n=922), AP2XI-2-A (n=2323). (B) Venn diagram shows the AP2XII-1 peaks con-ducted by Fan et al (AP2XII-1-F) , Antunes et al (AP2XII-1-A) , and this study (AP2XII-1-J). (C) Venn diagram shows the AP2XI-2 peaks conducted by Antunes et al (AP2XI-2-A) and this study (AP2XI-2-J).
