# Supplementary material for: AP2XII-1 and AP2XI-2 Suppress Schizogony Gene Expression in Toxoplasma gondii
Source: Int J Mol Sci. 2024 May 18;25(10):5527. doi: 10.3390/ijms25105527 (PMC11122372; doi:10.3390/ijms25105527)
Supplement: Supplementary file 1 [file ijms-25-05527-s001.zip › Figure S5.pdf]

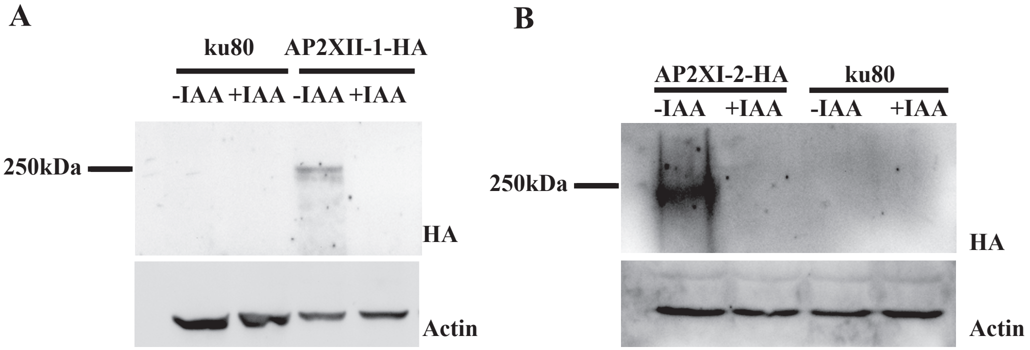

Figure S5. Western blot validation of the degradation of AP2XII-1 and AP2XI-2 proteins. RHΔku80, AP2XII-1-mAID (A) and AP2XI-2-mAID (B) parasites were treated with IAA or vehicle for 12 h, and subjected for immunoblotting using mouse anti-HA antibody. Actin was used as a loading control.
