# Supplementary material for: AP2XII-1 and AP2XI-2 Suppress Schizogony Gene Expression in Toxoplasma gondii
Source: Int J Mol Sci. 2024 May 18;25(10):5527. doi: 10.3390/ijms25105527 (PMC11122372; doi:10.3390/ijms25105527)
Supplement: Supplementary file 1 [file ijms-25-05527-s001.zip › Figure S6.pdf]

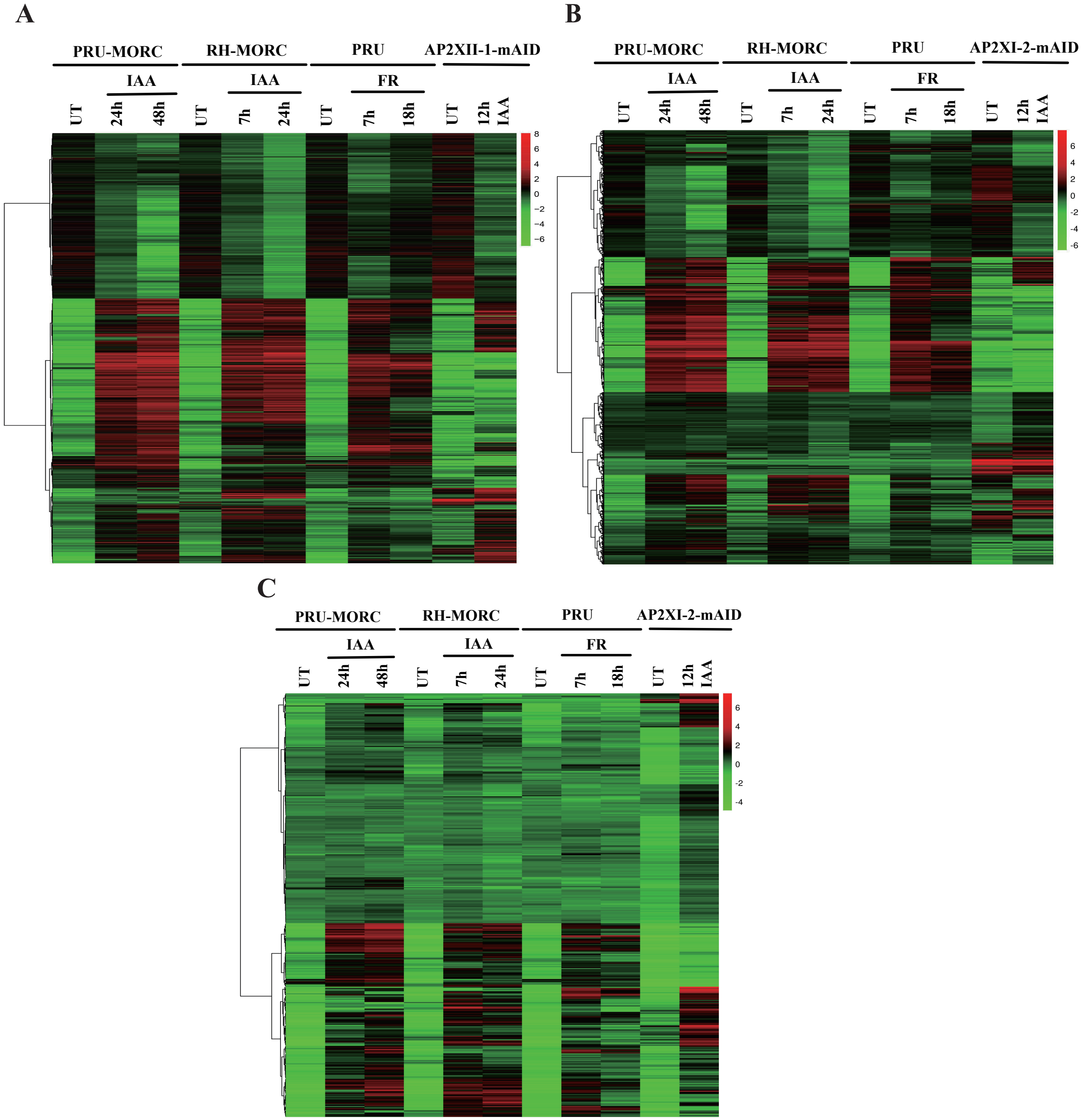

Figure S6. AP2XII-1 and AP2XI-2 downstream regulatory genes are consistent with MORC regulatory genes. Heatmaps showing differentially expressed genes after AP2XII-1 (A), AP2XI-2 (B) and MORC knockdown based on RNA-seq data. (C) Heatmap analysis of upregulated genes after AP2XI-2 and MORC knockdown.
