# Supplementary material for: AP2XII-1 and AP2XI-2 Suppress Schizogony Gene Expression in Toxoplasma gondii
Source: Int J Mol Sci. 2024 May 18;25(10):5527. doi: 10.3390/ijms25105527 (PMC11122372; doi:10.3390/ijms25105527)
Supplement: Supplementary file 1 [file ijms-25-05527-s001.zip › Figure S7.pdf]

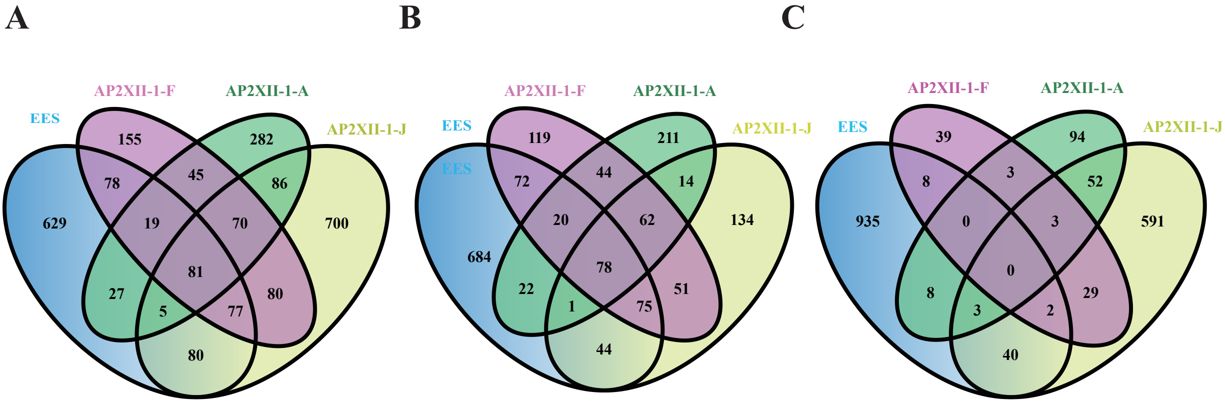

Figure S7. Highly overlap of differently expressed genes after AP2XII-1 knock-down in data from Fan et al , Antunes et al, and this study. The RNA-seq data from Fan et al, Antunes et al , and this study were compared, and the overall DEGs (A), upregulated genes (B), and downregulated genes (C) were overlapped by Venn diagrams. AP2XII-1-F, AP2XII-1-A, and AP2XII-1-J represents DEGs from Fan et al, Antunes et al, and this study, respectively.
