# Supplementary material for: AP2XII-1 and AP2XI-2 Suppress Schizogony Gene Expression in Toxoplasma gondii
Source: Int J Mol Sci. 2024 May 18;25(10):5527. doi: 10.3390/ijms25105527 (PMC11122372; doi:10.3390/ijms25105527)
Supplement: Supplementary file 1 [file ijms-25-05527-s001.zip › Figure S8.pdf]

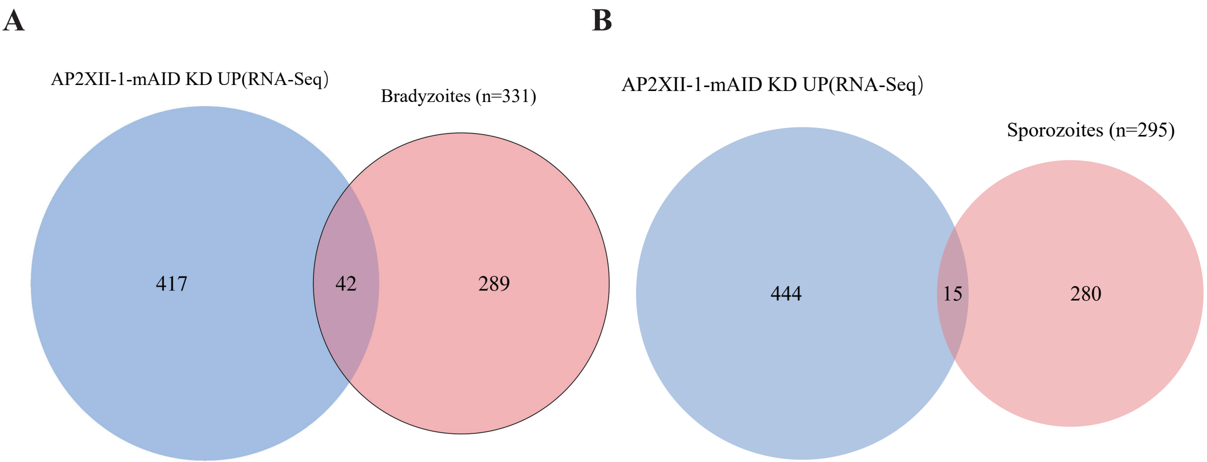

Figure S8. Transcription of a part of the bradyzoite and sporozoite-specific genes is affected after AP2XII-1 knockdown. (A) Overlap between up-regulated genes in AP2XII-1-mAID KD parasites and 331 genes exclusively expressed in bradyzoites. (B) Overlap between upregulated genes in AP2XII-1-mAID KD para-sites and 295 genes exclusively expressed in sporozoites.
