# Supplementary material for: AP2XII-1 and AP2XI-2 Suppress Schizogony Gene Expression in Toxoplasma gondii
Source: Int J Mol Sci. 2024 May 18;25(10):5527. doi: 10.3390/ijms25105527 (PMC11122372; doi:10.3390/ijms25105527)
Supplement: Supplementary file 1 [file ijms-25-05527-s001.zip › Figure S9.pdf]

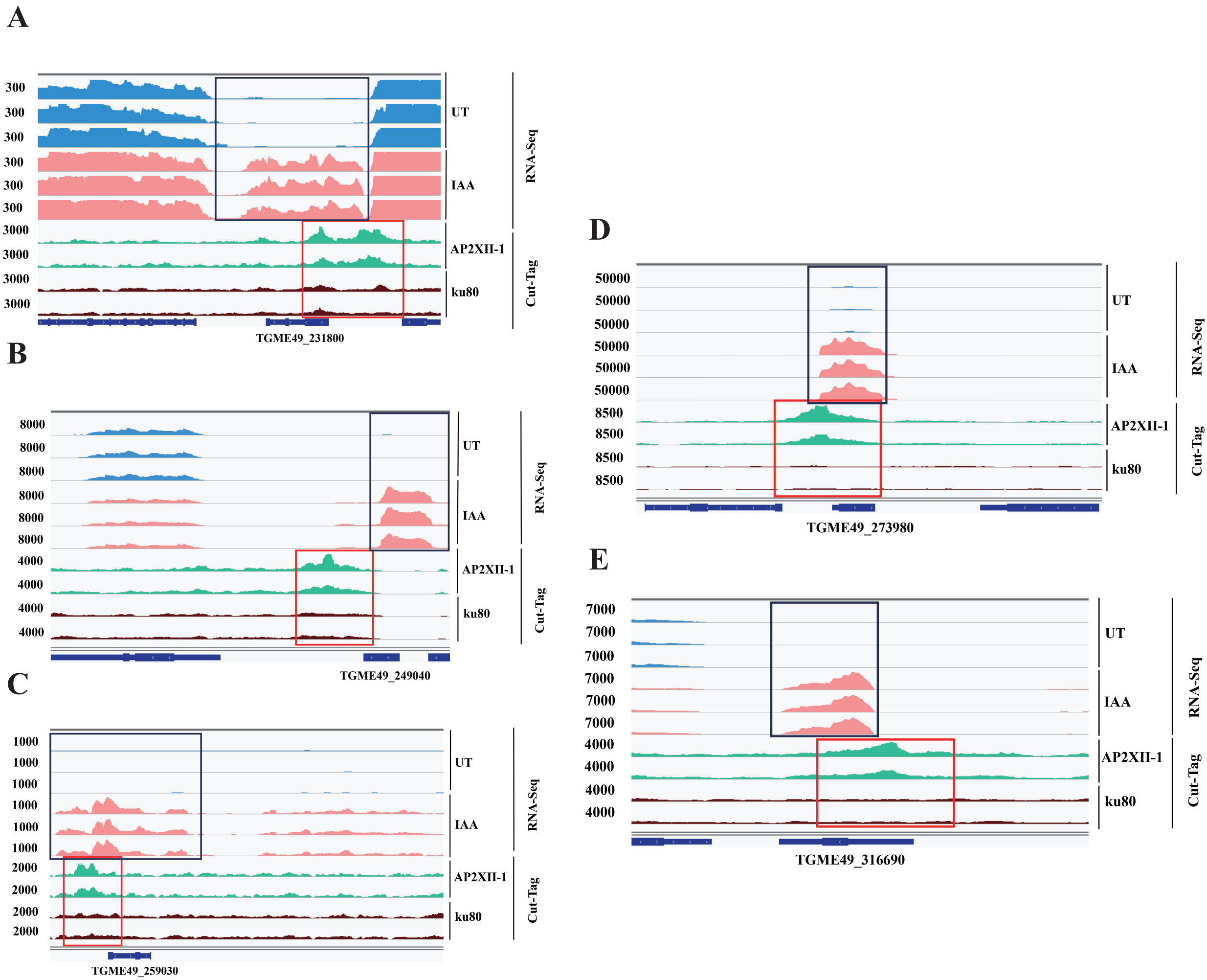

Figure S9. AP2XII-1 binds to the promoter of EES-specific genes and regulates their transcription. IGV screenshots of genomic regions of five EES-specific genes, in-cluding TGME49_249040 (A), TGME49_259030 (B), TGME49_273980 (C), TGME49_316690 (D) and TGME49_231800 (E). Cut-Tag profiles were obtained using antibodies directed against HA (ApiAP2XII-1 tagged) in chromatin sampled from the ApiAP2XII-1-mAID-HA strain and the Δku80 strain. RNA-seq data from ApiAP2XII-1-mAID-HA parasites after 12 h of treatment or no treatment with IAA are shown in red and blue peaks. The normalized RPKM for Cut-Tag and RNA-seq reads are shown on the y-axis.
